# Zeolite membrane reactors for efficient catalytic conversion of CO_2_ into high-value chemicals

**DOI:** 10.1093/nsr/nwag383

**Published:** 2026-06-19

**Authors:** Pengyao Yu, Soryong Chae, Chunzheng Wang, Xiao-Yun Li, Hailing Guo, Svetlana Mintova, Li-Hua Chen, Bao-Lian Su

**Affiliations:** State Key Laboratory of Heavy Oil Processing, China University of Petroleum (East China), Qingdao 266580, China; Laboratory of Inorganic Materials, University of Namur, Namur B-5000, Belgium; Department of Chemical and Environmental Engineering, University of Cincinnati, Cincinnati, OH 45221, USA; State Key Laboratory of Heavy Oil Processing, China University of Petroleum (East China), Qingdao 266580, China; State Key Laboratory of Silicate Materials for Architectures, Wuhan University of Technology, Wuhan 430070, China; State Key Laboratory of Heavy Oil Processing, China University of Petroleum (East China), Qingdao 266580, China; State Key Laboratory of Heavy Oil Processing, China University of Petroleum (East China), Qingdao 266580, China; Normandie Université, Laboratoire Catalyse et Spectrochimie (LCS), ENSICAEN, CNRS, Caen 14050, France; State Key Laboratory of Advanced Technology for Materials Synthesis and Processing, Wuhan University of Technology, Wuhan 430070, China; Laboratory of Inorganic Materials, University of Namur, Namur B-5000, Belgium; State Key Laboratory of Advanced Technology for Materials Synthesis and Processing, Wuhan University of Technology, Wuhan 430070, China

**Keywords:** zeolite membrane reactors, carbon capture and utilization, CO_2_ catalytic conversion, high-value chemicals, thermodynamic constraints

## Abstract

Converting CO_2_ into high-value chemicals is vital for achieving carbon capture and utilization. Although many advanced catalysts have been developed to activate the thermodynamically stable CO_2_ molecules, overcoming equilibrium limitations remains essential for improving conversion efficiency. Zeolite membrane reactors (ZMRs) have been explored to overcome thermodynamic constraints and further improve conversion efficiency. This review provides insights into catalyst evolution and highlights the pivotal role of zeolite membranes in enhancing CO_2_ conversion to high-value chemicals such as methanol, dimethyl ether and dimethyl carbonate within ZMRs. An overview of zeolite membrane selection, preparation and catalyst-membrane coupling strategies is presented, along with recent advances in process modeling to elucidate reaction–separation interactions. The stability of ZMR systems under practical conditions is also discussed. Finally, future perspectives, identifying key challenges and potential research directions, are presented. This review aims to inspire further innovations in efficient CO_2_ conversion to high-value chemicals using ZMRs.

## Teaser

This review highlights ZMRs as a promising approach for overcoming equilibrium limitations and advancing the efficient conversion of CO_2_ into high-value chemicals.

## INTRODUCTION

**Figure 1.**
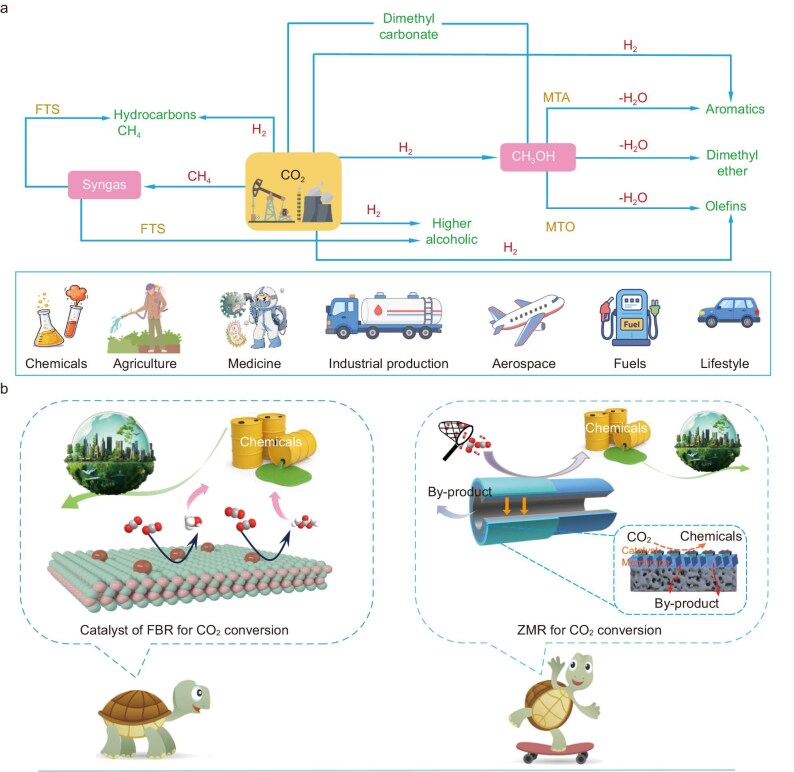
(a) The routes of CO_2_ catalytic conversion and (b) the comparison of FBRs and ZMRs. FTS, Fischer–Tropsch process.

The rapid industrialization has led to the significant release of CO_2_ into the atmosphere, leading to climate change and environmental degradation [1,2]. To address this challenge, sustainable CO_2_ management through carbon capture and utilization has emerged as a critical area of research and innovation [3,4]. One promising approach is the use of CO_2_ as a renewable feedstock for producing high-value-added chemicals [5–10]. Extensive research efforts have been devoted to catalyzing CO_2_ conversion into high-value fuels and chemicals such as methanol (CH_3_OH), formic acid (HCOOH), dimethyl carbonate (DMC), dimethyl ether (DME), light olefins, syngas and aromatic hydrocarbon [3,5] (Fig. 1a).

Significant advancements have been made in developing CO_2_ catalytic conversion [11], employing a variety of approaches such as thermochemical [12–14], electrochemical [15], photochemical [16] and biochemical [17] methods. In particular, thermochemical conversion has received attention due to its maturity, scalability and compatibility with existing chemical processes. However, CO_2_ catalytic conversion reactions inherently face several persistent challenges: (i) the intrinsic difficulty in activating stable CO_2_ molecules [18,19], (ii) the limitation imposed by thermodynamic equilibrium on the reaction process [20] and (iii) the formation of by-products that reduce product selectivity or lead to catalyst deactivation [18,20]. Catalytic design strategies, including the incorporation of vacancies, defects and interfaces, have greatly enhanced the activation of CO_2_ molecules, driving remarkable progress in CO_2_ catalytic conversion processes. For instance, an advanced catalyst, sulfur vacancy-rich MoS_2_ for CO_2_ hydrogenation to methanol has been reported to achieve a CO_2_ conversion rate of 12% and methanol selectivity of 94.3% at 180°C [13]. However, the issues of thermodynamic equilibrium limitations and the adverse effects of by-products remain restrict the efficient conversion of CO_2_.

Selectively removing reaction products not only drives the equilibrium toward the product side but helps mitigate catalyst deactivation [21,22]. Two primary strategies can be employed to address these issues: (i) utilizing organic molecules, zeolite materials or specialized devices to shift the equilibrium by selectively removing by-products [22] and (ii) further converting intermediate products into other high-value chemicals, which simultaneously promotes the desired primary reaction. Recent studies have demonstrated the potential of zeolite tandem catalysts for converting CO_2_ into olefins or aromatics [18]. For example, a representative catalyst, ZnZrO*_x_*/Zeolite Socony Mobile No. 5 (ZSM-5) tandem catalyst, has been employed to couple CO_2_ hydrogenation with the subsequent methanol-to-aromatics (MTA) reaction, achieving a CO_2_ conversion rate of over 20% with a selectivity of 90% [23]. Despite above promising developments, the broader application of such equilibrium-manipulating approaches remains limited. These methods often face challenges such as the regeneration or replacement of adsorbents, and the consumption of intermediates still fails to completely eliminate equilibrium constraints or by-product effects. Therefore, there is an urgent need for an efficient strategy to convert CO_2_ into high-value chemicals.

A membrane reactor is a system that integrates membrane separation with a reaction process, offering a promising approach to improve reaction efficiency [24–26]. Compared with fixed-bed reactors (FBRs), membrane reactors integrate the catalyst used in conventional fixed-bed systems with a separation membrane, enabling the simultaneous reaction and selective removal of by-products through the membrane. This makes them particularly suitable for CO_2_ catalytic conversion reactions. Successfully applying membrane reactors to CO_2_ catalytic conversion requires the simultaneous advancement of high-performance catalytic and separation modules. In this context, various efficient catalysts such as MoS_2_, metal oxide, metal and metal alloy have been designed and optimized for CO_2_ catalytic conversion processes [27,28]. Naturally, these catalysts, which have been well established in fixed-bed systems, can also be directly utilized as catalytic modules in membrane reactors. However, membrane reactors for CO_2_ catalytic conversion impose stringent requirements on the separation membrane. The membrane must withstand harsh reaction conditions and possess appropriate hydrophilicity or small pore sizes to enable the selective removal of by-products such as water or hydrogen. Therefore, the selection of separation membrane is crucial for achieving efficient CO_2_ catalytic conversion in membrane reactors.

Distinct from the limited chemical stability of polymeric membranes and the broad pore size distribution of conventional inorganic membranes, zeolite membranes are well recognized for their excellent chemical and hydrothermal stability, tunable hydrophilicity/hydrophobicity, exceptional mechanical stability and well-defined pore structure, making them widely regarded as one of the most promising membrane materials for CO_2_ conversion applications [29–34]. As shown in Fig. 1b, an FBR conducts catalytic reactions in a stationary catalyst bed, whereas in a zeolite membrane reactor (ZMR), the catalyst and separation membrane are integrated into a single system, allowing continuous removal of by-products during the reaction. Compared to FBR, ZMR are promising candidate for overcoming thermodynamic limitations, promoting target reactions, removing by-product to prevent catalyst deactivation and achieving improved conversion efficiency and product selectivity [29,32,35]. Notably, a high conversion rate of 61% has been achieved in a water-conduction ZMR for CO_2_ hydrogenation to methanol, providing strong evidence of the potential of ZMR in CO_2_ catalytic conversion applications [36].

ZMRs have introduced a significant technological innovation to the CO_2_ catalytic conversion process, effectively maximizing CO_2_ conversion efficiency. This review comprehensively summarizes recent advances in the application of ZMRs for CO_2_ catalytic conversion and highlights the key challenges and opportunities in this emerging field. It first traces the development of CO_2_ conversion technologies from traditional catalysts to zeolite tandem catalysts and ultimately to ZMRs, emphasizing the transition of research focus from enhancing catalytic activity to breaking thermodynamic equilibrium limitations, and underscoring the crucial role of membrane reactors in overcoming these constraints. Furthermore, the review discusses the design, fabrication, the state of the art and challenge of zeolite membranes in ZMRs tailored to different reaction characteristics, emphasizing the pivotal role of selective separation in promoting catalytic efficiency. It summarizes the structural tunability and various configurations of ZMRs, and compares their superior performance in terms of conversion efficiency, product selectivity and stability with conventional FBRs. It also reviews relevant process simulation studies and recent advances, with a particular focus on the advantages of ZMRs in performance enhancement as well as the associated structural and process parameters. Finally, it provides perspectives on future directions and conclusive insights into the development of ZMR technologies.

It is important to note that most previous reviews on CO_2_ conversion membrane reactors or zeolite membranes have primarily focused on either catalyst development, membrane fabrication strategies or separation performance as independent topics. In contrast, our present review adopts a system-level and reaction engineering-oriented perspective, integrating catalyst design, zeolite membrane microstructure and reactor configuration into a unified framework. Furthermore, beyond summarizing material advances, this review highlights the importance of reaction–separation coupling, rate matching and reactor design as key principles for process intensification. By bridging the gap between material science and reactor engineering, this review provides a more comprehensive and mechanistic understanding of ZMRs for CO_2_ conversion, offering insights that extend beyond conventional catalyst or membrane centered analyses and aiming to guide the rational design of next-generation membrane reactor systems.

## DEVELOPMENT OF CATALYSTS FOR CO_2_ CONVERSION

In recent years, significant progress has been made in the field of sustainable CO_2_ catalysis and catalyst development [3,5,7,28,37–39]. Research has primarily focused on understanding the nature of active sites, including vacancy engineering, adsorption, activation and acid–base properties, while optimizing catalyst supports to enable advanced surface engineering, spatial confinement and the incorporation of suitable additives [40,41]. These advancements are reviewed in the context of their optimal performance in current research, along with perspectives on existing challenges [1]. This review shifts the emphasis from the detailed design of active sites and the interaction between the active phase and the support to the broader developmental trends in catalyst design. The goal is to highlight strategies that can guide the future integration of these catalysts into advanced membrane reactors.

**Figure 2.**
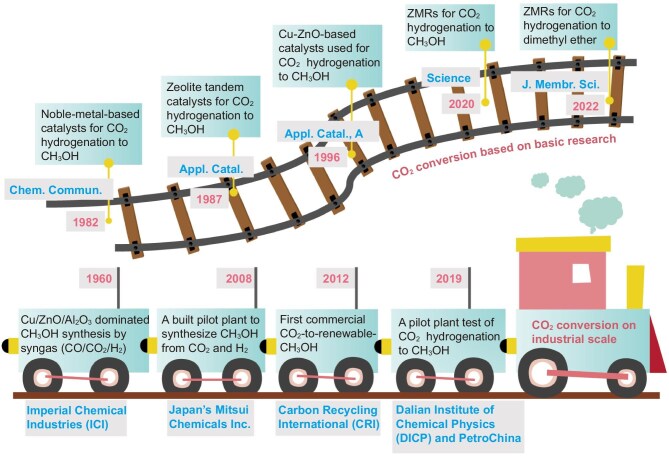
The timeline of main developments in the field of CO_2_ catalytic conversion.

As illustrated in Fig. 2, Cu/ZnO/Al_2_O_3_ catalysts have dominated industrial methanol synthesis from syngas (containing CO_2_) since the 1960s. Subsequently, research expanded to include methanol synthesis from syngas mixed with CO_2_ hydrogenation. In the 1980s, Kiennemann *et al.* [42] developed noble-metal-based catalysts for CO_2_ hydrogenation to methanol, ushering in the era of pure CO_2_ hydrogenation. Since the 1990s, Cu/ZnO-based catalysts have been used directly for CO_2_ hydrogenation to methanol, paving the way for industrialization of this reaction [43]. In 2008, Japan’s Mitsui Chemicals, Inc. built a pilot plant of CO_2_ hydrogenation to methanol, achieving an annual production capacity of ∼100 metric tons [7]. Later, in 2012, Carbon Recycling International became the first to achieve industrial-scale CO_2_-to-renewable-methanol production, leveraging Iceland’s abundant geothermal resources [7]. Significant progress has also been made in other regions lacking Iceland’s renewable energy advantages. For instance, in 2019, PetroChina collaborated with the Dalian Institute of Chemical Physics (DICP) on a pilot plant project for CO_2_ hydrogenation to methanol. In 2020, DICP, in partnership with the Lanzhou New Area Petrochemical Industry Investment Group, successfully executed a 1000-ton industrial demonstration project, marking another milestone in the field [7].

In addition to methanol, converting CO_2_ into a variety of other high-value chemicals is also a key area of research. To date, CO_2_ has been converted into a range of chemicals, including methanol, ethanol, higher alcohols, hydrocarbons, olefins, aromatics, carboxylic acids, carbonates, ethers and gasoline. Notably, Fujimoto and Shikada [44] were the first to propose mixed catalysts composed of Cu-based catalysts for methanol synthesis and zeolite catalysts for C_2_–C_5_ hydrocarbons, which laid the foundation for the development of tandem catalysts that use methanol as an intermediate. These tandem catalysts consist of two components: a methanol synthesis catalyst and a solid acid catalyst for the conversion of methanol. Zeolites are particularly well-suited as solid acid catalysts due to their unique pore structures, shape selectivity, tunable acidity, controllable hydrophilicity and exceptional (hydro)thermal stability. These properties enable tandem catalysts to direct hydrogenation reactions into different products by leveraging variations in pore size and acidity across different zeolite types. The process begins with CO_2_ hydrogenation to methanol, followed by methanol conversion via methanol-to-olefins (MTO), MTA and methanol condensation reactions, which collectively drive the reaction equilibrium [45].

While tandem catalysis can effectively couple sequential reactions and improve overall selectivity, it is inherently constrained by thermodynamic equilibrium, as reaction intermediates and products remain within the same reaction environment. To drive the thermodynamic equilibrium and enhance reaction efficiency, membrane reactor based on Le Chatelier’s principle has been proposed. In 2020, a ZMR has been successfully used for CO_2_ hydrogenation to methanol [36], and later, in 2022, for CO_2_ hydrogenation to DME [46]. Water-conducting membrane enabled highly efficient *in situ* by-product removal, significantly enhancing CO_2_ conversion and chemicals yield.

These reactions are often limited by thermodynamic equilibrium and are accompanied by side reactions, which significantly affect product selectivity and conversion efficiency. To promote the desired reactions and increase CO_2_ conversion rates, it is crucial to shift the equilibrium in a controlled manner. From the use of zeolite-based tandem catalysts to the development of ZMRs, researchers have made significant progress by promoting equilibrium conversion through the further consumption of products or the removal of by-products. However, it has become increasingly clear that zeolite-based tandem catalysts by themselves are insufficient to fully satisfy the demands of these reactions.

On one hand, the application scope of tandem zeolite catalysts is relatively limited and cannot be universally applied to all reaction systems. On the other hand, the presence of water vapor in the reaction environment accelerates catalyst sintering and deactivation, further complicating the process. Although tandem zeolite catalysts can promote CO_2_ conversion by consuming products, they cannot effectively eliminate the detrimental influence of by-products.

In this context, the timely removal of by-products not only promotes the equilibrium reaction in the forward direction but also mitigates the poisoning effect of water vapor on the catalyst. Engineering innovations may offer solutions to overcome the challenges faced by catalysts in these processes. ZMR presents a promising approach to overcome the equilibrium limitations in CO_2_ catalytic conversion. Therefore, this review focuses on summarizing the progress of ZMRs for CO_2_ conversion, with particular attention to the construction of ZMRs and the use of zeolite membranes in these systems.

## ZMRS FOR CATALYTIC CONVERSION OF CO_2_ INTO HIGH-VALUE CHEMICALS

Membrane reactor technology, which integrates catalytic reactions and membrane separation, offers a promising method to shift the equilibrium by removing by-products in a timely manner [47]. Typically, CO_2_ conversion reactions are carried out under high temperature and pressure conditions. Therefore, the membrane used in these reactors must meet following two key criteria: (i) possessing high thermal and chemical stability to endure the harsh reaction conditions, and (ii) allowing steam permeation while effectively blocking other gases, thereby enhancing both selectivity and durability [20]. Zeolite membranes, characterized by superior chemical/hydrothermal stability, tunable hydrophilicity, great acidic property and ordered small pore structure (<1 nm), are considered the most attractive materials for membrane reactors for CO_2_ conversion [48]. The following sections will systematically discuss ZMRs from several key perspectives, including the factors governing zeolite membrane performance in ZMRs, the current research status of zeolite membranes for ZMR applications, the remaining challenges and corresponding strategies, the configurations of ZMRs and their performance advantages.

### Key factors governing the performance of zeolite membranes in ZMRs

The reaction performance and long-term stability of ZMRs are closely correlated with the water/gas permeation properties of zeolite membranes [29]. These performance characteristics are governed by the synergistic effect of several key parameters, including zeolite topology, Si/Al ratio (SAR), extra-framework cations and membrane quality. Together, these factors determine the effective pore size, hydrophilicity, adsorption behavior and structural stability of the membrane, thereby directly influencing the efficiency of *in situ* water removal and the overall stability of the reactor. In the following section, zeolite membranes suitable for CO_2_ catalytic conversion in membrane reactors are systematically discussed from several key aspects, including the selection of framework topology based on pore structure and molecular sieving properties, the optimization of the SAR to balance hydrophilicity and hydrothermal stability, the role of extra-framework cations in regulating adsorption and transport behavior and the influence of membrane fabrication quality on overall performance.

#### Framework structure

The intrinsic framework structure of zeolites defines their pore size, diffusion pathways and molecular sieving properties [38], which plays a decisive role in governing water/gas separation performance in ZMRs. Unlike conventional separation processes, ZMRs for CO_2_ catalytic conversion require membranes that can selectively and continuously remove water from complex gas mixtures under elevated temperature and pressure. Therefore, the precise matching between zeolite apertures and molecular sizes becomes a critical factor in both separation efficiency and reactor stability.

Zeolite frameworks with small pore apertures, such as Linde type A (LTA; ∼0.4 nm), sodalite (SOD; ∼0.28 nm) and chabazite (CHA; ∼0.38 nm) type, are particularly effective for water-selective separation due to their comparable pore sizes to the kinetic diameter of water molecules (∼0.26 nm) [49]. These structures enable strong molecular sieving effects, allowing preferential permeation of water while excluding larger molecules such as CO_2_ and methanol. In contrast, medium-pore zeolites such as mobil five (MFI) (∼0.51 nm) and larger-pore frameworks like faujasite (FAU; ∼0.74 nm) exhibit weaker size exclusion effects but offer enhanced diffusivity and tunable selectivity [49]. Their larger pore sizes may lead to reduced water/gas selectivity, necessitating additional control through framework composition or cation modification.

In summary, the pore structure and framework topology of zeolite membranes fundamentally determine the feasibility of water/gas separation in membrane reactors. However, framework topology alone is insufficient to fully describe membrane behavior. The actual separation performance also strongly depends on the chemical composition of the framework, particularly the SAR and associated cations. In addition, membrane quality is a critical factor in achieving superior separation performance and ensuring the stable operation of membrane reactors.

#### Si/Al ratio

The SAR is a key structural parameter that controls the polarity and hydrophilicity of zeolite frameworks. A lower SAR corresponds to a higher aluminum content, which introduces more negative framework charges that must be balanced by extra-framework cations [50]. This results in stronger electrostatic interactions with polar molecules such as H_2_O, thereby significantly enhancing water adsorption and perm-selectivity.

In ZMRs for CO_2_ hydrogenation, such strong hydrophilicity is highly desirable because it enables efficient *in situ* water removal. For example, low-silica LTA and FAU membranes typically exhibit excellent water selectivity in ZMRs. However, a high Al content introduces an inherent trade-off between hydrophilicity and structural stability in zeolite membranes. While increased Al incorporation enhances hydrophilicity, it simultaneously weakens the hydrothermal stability of the zeolite framework under high-temperature, water-rich conditions [51]. In addition, high-Al zeolites exhibit strong electronegativity, which can lead to significant intercrystalline repulsion during membrane growth, thereby promoting the formation of intercrystalline defects [50]. These defects markedly deteriorate separation performance and compromise the long-term stability of the membrane.

Therefore, in the design of high-quality zeolite membranes for ZMRs, it is essential to carefully tune the SAR to achieve an optimal balance between hydrophilicity and structural stability. Meanwhile, advanced fabrication strategies for high-Al zeolite membranes should be rationally developed to minimize intercrystalline defects, thereby enhancing both membrane performance and the operational lifetime of membrane reactors.

#### Extra-framework cations

Extra-framework cations not only balance the framework charge of zeolites but also play a crucial role in regulating the effective pore size and adsorption behavior of zeolite membranes. Studies have shown that in hydrophilic zeolite membranes (e.g. LTA membranes), cations such as Na^+^ are located within the cavities, acting as ‘gate-controlled’ sites that reduce the effective pore aperture and enhance electrostatic interactions with polar molecules [36]. This results in strong preferential adsorption of water and facilitates its selective transport through the membrane. Density functional theory (DFT) studies have further shown that the interaction between water molecules and cations significantly lowers the energy barrier for water adsorption, thereby enhancing separation performance [36]. For example, Na^+^-exchanged zeolites exhibit a strong affinity toward polar molecules such as H_2_O, with the H_2_O/H_2_ separation factor increasing from ∼1 for the parent H-ZSM-5 membrane to 22 after Na^+^ exchange [52].

In addition, cation exchange provides an effective means to regulate the pore size of zeolite membranes. For example, replacing Na^+^ with larger monovalent cations (e.g. K^+^ and Ag^+^) can reduce the effective pore aperture and enhance molecular sieving selectivity [53], while multivalent cations (e.g. Cu^2+^) can increase the membrane’s affinity toward water and modify its adsorption behavior. For instance, Cu^2+^-exchanged mordenite (MOR) membranes exhibit enhanced hydrophilicity and a reduced effective pore size, leading to improved H_2_O selectivity in CO_2_ hydrogenation systems [54].

However, the presence of cations may also influence membrane stability. Cation migration or leaching may occur under high temperature and pressure conditions, resulting in alterations of the pore structure and a gradual decline in separation performance over time. Therefore, the stability of extra-framework cations is a critical factor for ensuring the long-term operation of ZMRs.

#### The quality of zeolite membranes

Beyond framework topology and composition, the quality of zeolite membranes is a decisive factor governing their water/gas separation performance and long-term stability in ZMRs. Membrane quality is typically defined by structural features such as intercrystalline defects, grain boundary integrity and thickness uniformity, etc., all of which directly influence molecular transport behavior and separation selectivity [29].

Among these factors, the presence of defects is particularly critical. Non-selective defects, including microcracks, pinholes and poorly intergrown grain boundaries, will lead to a loss of molecular sieving capability and a significant decline in water/gas selectivity. In the context of CO_2_ catalytic conversion, such defects will result in insufficient water removal, thereby weakening the reaction–separation coupling and accelerating catalyst deactivation [29]. Therefore, achieving defect-free and highly intergrown membranes is essential for maintaining high separation efficiency and stable reactor operation.

Membrane thickness also plays a crucial role in determining permeation performance. Thinner membranes reduce diffusion resistance and enable higher permeation, which is beneficial for rapid product removal in ZMRs. However, excessively thin membranes are more susceptible to defect formation and mechanical instability under high temperature and thermal conditions. Conversely, thicker membranes generally exhibit improved structural robustness but suffer from lower permeation, limiting their effectiveness in *in situ* separation. This highlights an inherent trade-off between permeability and mechanical stability, requiring precise control over membrane thickness during synthesis.

In summary, membrane quality fundamentally determines whether the intrinsic separation properties of zeolite frameworks can be effectively translated into reactor performance. The fabrication of defect-free and well-grown zeolite membranes is essential for achieving high water/gas selectivity, rapid permeation and long-term stability in ZMRs for CO_2_ catalytic conversion.

### Current research progress on zeolite membranes for CO_2_ catalytic conversion in ZMRs

**Figure 3.**
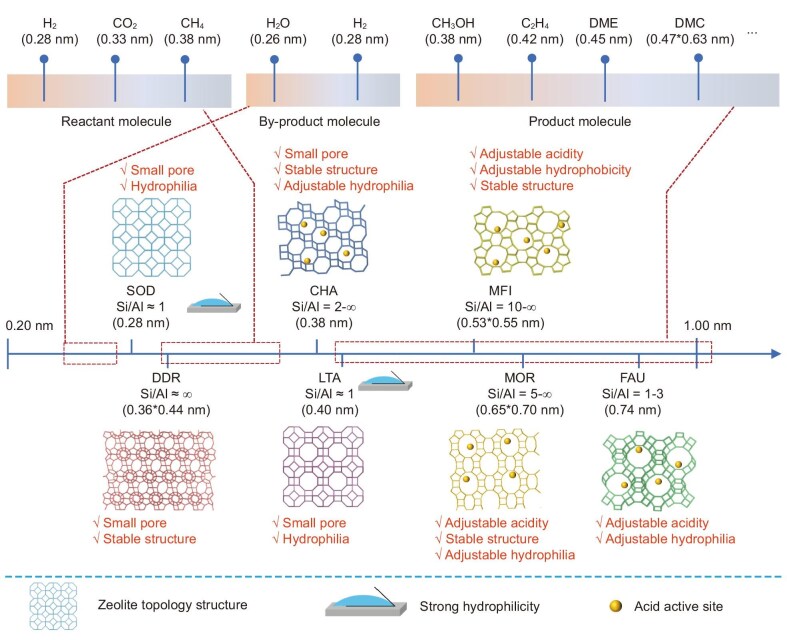
Schematic illustration of key properties of zeolite membranes for ZMR applications.

This section summarizes the current research status of zeolite membranes that have been applied or show potential for application in ZMRs. Figure 3 highlights the relationship between typical reactant/product molecules and the ideal pore size of zeolite membranes, as well as key properties that critically influence their performance in ZMRs, including acidity, SAR and hydrophilicity.

It is worth noting that zeolite membranes with high Al content generally exhibit strong hydrophilicity. Therefore, low-silica zeolites such as SOD and LTA typically possess intrinsically high hydrophilicity that can be directly utilized for water-selective separation. In contrast, for zeolite membranes such as FAU, CHA, MFI and MOR, hydrophilicity can be tuned by adjusting the SAR. Moreover, this tunability also imparts catalytic functionality, particularly in terms of acidity. The acidity discussed here mainly refers to Brønsted acid sites, which can provide active sites required for reactions such as CO_2_ hydrogenation and subsequent transformations. For example, H-FAU and H-ZSM-5 membranes can facilitate methanol dehydration to DME while enabling *in situ* water removal. Similarly, CHA (e.g. SAPO-34) and H-MOR membranes have been widely used in MTO and DME carbonylation reactions, respectively, demonstrating their potential in tandem processes following CO_2_ hydrogenation [55].

The following section will provide a detailed discussion of the characteristics and current progress of zeolite membranes that have been applied or show promise for use in ZMRs, along with guidelines for selecting suitable membranes based on specific reaction requirements. Table 1 summarizes the water/gas separation performance of membrane materials that have been successfully applied or exhibit strong potential in ZMR systems.

**Table 1.** A summary of the zeolite used for the membrane reactors for CO_2_ catalytic conversion.

Membrane	Temperature	Pressure	Water permeance (mol m^−2^ s^−1^ pa^−1^)	Separation selectivity (mixture)	Ref.
LTA	250°C	2.1 MPa	1.49 × 10^−7^	H_2_O/CO_2_ 10 722	[36]
LTA	260°C	0.1 MPa	1.13 × 10^−7^ 1.11 × 10^−7^ 1.15 × 10^−7^	H_2_O/CO_2_ 55.4 H_2_O/H_2_ 34.1 H_2_O/CH_3_OH 258.1	[56]
LTA	200°C	2 MPa	8.57 × 10^−7^	H_2_O/CO_2_ 5376 H_2_O/H_2_ 1515 H_2_O/CH_3_OH 183	[57]
SOD	200°C	0.1 MPa	6.59 × 10^−8^	H_2_O/CO_2_ 22.6 H_2_O/H_2_ 4.6 H_2_O/CH_3_OH 233 H_2_O/DME 253	[58]
H-ZSM-5	280°C	0.1 MPa	9.64 × 10^−8^	H_2_O/CO_2_ 61.4 H_2_O/H_2_ 20.6 H_2_O/DME 128.3	[46]
FAU	180°C	5 MPa	2.1 × 10^−7^	H_2_O/H_2_ 59 H_2_O/CH_3_OH 9.0	[59]
CHA	200°C	2.0 MPa	2.4 × 10^−7^	H_2_O/H_2_ 370 H_2_O/CO_2_ 930	[60]
MOR	180°C	0.1 MPa		H_2_O/CO_2_ < 2 H_2_O/H_2_ < 1	[35]
MOR	250°C	0.1 MPa	6.11 × 10^−8^ 6.34 × 10^−8^ 6.18 × 10^−8^	H_2_O/CO_2_ 17.2 H_2_O/H_2_ 8.4 H_2_O/CH_3_OH 21.2	[54]

LTA zeolite membranes display exceptional substantial separation performance for water/gas and organic/H_2_O mixture due to their strong hydrophilicity and small pore size (∼0.4 nm). This advantage has enabled its industrial application in organic/H_2_O separation, facilitating the development of water-permeable membrane reactors [50,61]. Notably, LTA membranes have been successfully implemented in ZMRs for CO_2_ hydrogenation to methanol and DME [56,62].

LTA zeolite membranes, functioning as Na^+^-gated water-conduction membrane (WCM), have demonstrated remarkable water/gas separation performance in ZMRs for methanol production. These membranes show a high H_2_O/CO_2_ selectivity of 10 722, with a water permeability of 1.49 × 10^−7^ mol m^−2^ s^−1^ pa^−1^ under the conditions of 210°C and 21 bar [36] (Table 1). The positioning of Na^+^ ions within the LTA nanocavities at three distinct sites decreases the effective aperture size. DFT supports the observation on the strong interaction between polar H_2_O molecules and Na^+^ ions [36].

Furthermore, oriented LTA membranes have been used as part of ZMRs for separation during CO_2_ hydrogenation to methanol. These ZMRs, utilizing LTA membranes combined with Cu-ZnO-Al_2_O_3_-ZrO_2_, showed separation factors of 258.1, 55.4 and 34.1 for H_2_O/CH_3_OH, H_2_O/CO_2_ and H_2_O/H_2_, respectively [56]. Additionally, Kawi *et al.* [57] fabricated a semi-hollow LTA membrane with high water permeability for stimulated CO_2_ hydrogenation to methanol.

SOD zeolite membranes, characterized by their higher framework density, offer superior chemical and thermal stability compared to LTA zeolite membranes [58]. With their high hydrophilicity, smaller pore size (∼0.28 nm) and stable framework structure, SOD membranes are suitable for by-product removal under harsh reaction conditions. A tubular SOD membrane has been successfully applied to selectively remove water during esterification reactions, demonstrating stable water pervaporation performance even at pH above 2.9 [63].

Wang *et al.* [58] studied the separation performances of SOD membranes in the separation of gas mixtures including H_2_O/CH_3_OH, H_2_O/DME and H_2_O/DMC at 125°C–200°C. The study results emphasized their ability to effectively remove H_2_O due to a combination of hydrophilicity and molecular sieving. Simulation results on an SOD membrane reactor for CO_2_ conversion to DME revealed minimum H_2_O/H_2_ selectivity and H_2_O permeability of 1.5 and 1.49 × 10^−7^ mol m^−2^ s^−1^ pa^−1^, respectively [64]. These attributes underscore the suitability of SOD membranes for synthesizing CH_3_OH, DME and DMC through CO_2_ conversion in ZMRs. Although their water permeance is typically lower due to their smaller pore size, the trade-off in performance makes them ideal for applications requiring high selectivity and stability under harsh conditions.

Due to its intrinsic hydrophilicity and precisely defined pore size (0.38 nm), CHA zeolite membrane enables accurate recognition and transport of water molecules, making it highly suitable for applications such as solvent dehydration and seawater desalination via pervaporation [65]. Compared with LTA, SOD and FAU types, CHA membranes are high-silica zeolites with excellent structural stability. Moreover, Huang *et al.* [60] incorporated hydrogenation catalysts into a 19-channel monolithic CHA membrane to successfully fabricate a large-area ZMR for CO_2_ hydrogenation to methanol. The large-area CHA membrane exhibited excellent vapor–gas separation performance, with H_2_O/CO_2_ and H_2_O/H_2_ separation factors of 930 and 370, respectively. The successful application and scale-up of CHA membranes in CO_2_ hydrogenation provide a feasible pathway for the industrial development of ZMRs.

ZSM-5 zeolite (∼0.51 nm) with MFI-type framework structure has hydrophilic and acidic properties that can be regulated according to the silica–aluminium content. H-ZSM-5 membranes provide acidic active sites that facilitate methanol dehydration to DME while offering dehydration pathways for by-products, demonstrating great potential for direct DME production from CO_2_ hydrogenation [46,66]. Their high water/gas selectivity—H_2_O/DME = 128.3, H_2_O/CO_2_ = 61.4 and H_2_O/H_2_ = 20.6—effectively shifts the thermodynamic equilibrium, significantly enhancing CO_2_ conversion and DME selectivity (Table 1). Additionally, simulation results further confirm the suitability of ZSM-5 membranes for CO_2_ conversion to DME [46]. Its molecular selectivity and tunable hydrophilic as well as acidic properties will lay a solid foundation for the application of ZSM-5 membranes in ZMR.

FAU zeolite membranes (∼0.74 nm) with 12-membered rings have been utilized for pervaporation in dehydration applications [67]. Their separation performance has been demonstrated in mixtures of CH_3_OH, H_2_ and H_2_O under high-temperature (i.e. 180°C) and high-pressure (i.e. 5 MPa) conditions, achieving a separation factor of 59 for H_2_O/H_2_ [59] (Table 1). Huang *et al.* [68] evaluated the pervaporation performance of FAU zeolite membranes for a 90 wt% DMC/H_2_O mixture, achieving a flux of 3.6 kg m^−2^ h^−1^ with a remarkable separation factor exceeding 10 000. Additionally, the acidic sites of FAU membranes impart catalytic properties, making them highly promising candidates for water-permeable membrane reactors. These reactors are particularly suitable for high-temperature and high-pressure CO_2_ conversion processes, including the catalytic conversion of CO_2_ to methanol, DME and DMC.

MOR zeolite membranes (0.65 × 0.7 nm) with optimized SARs and acid resistance and hydrophilicity, showing promise in applications such as acetic acid dehydration and esterification reactions via pervaporation dehydration [69]. Compared with high-Al-content zeolite membranes such as LTA, MOR membranes possess superior structural stability, making them promising candidates for ZMRs for CO_2_ catalytic conversion. Menendez *et al.* [35] compared the separation performance of MOR and LTA zeolite membranes in H_2_O/H_2_/CO_2_ mixtures, reporting lower separation factors for MOR membranes (H_2_O/CO_2_ and H_2_O/H_2_). We believe this is due to the relatively large pore size of the MOR membranes, resulting in very low molecular sieving selectivity for gas separation. Huang *et al.* improved the hydrophilicity of MOR membranes and reduced their intrinsic pore size by Cu^2+^ ion exchange, achieving separation factors of 17.2, 8.4 and 21.2 for H_2_O/CO_2_, H_2_O/H_2_ and H_2_O/CH_3_OH, respectively. The excellent water–vapor separation performance enabled their successful application in ZMRs for CO_2_ hydrogenation to methanol [54].

Other zeolite membranes also have been investigated for separation purposes, demonstrating their potential applications in ZMRs for CO_2_ conversion. Similarly, Lee *et al.* achieved the production of DME from methanol in a BEA membrane reactor, demonstrating the excellent dehydration capability of the BEA zeolite membrane [70]. In addition, several other zeolite membranes have been widely reported for use in ZMRs. Specifically, Choi *et al.* [71] reported the use of deca-dodecasil 3R (DDR) membranes with H_2_-selective for methylcyclohexane dehydrogenation in a ZMR. Kawi *et al.* [72] developed a SAPO-34 hydrogen-permeable membrane reactor for propane dehydrogenation. Although these membranes have not yet been widely applied in the field of CO_2_ conversion, their ability to selectively separate H_2_ suggests strong potential for future applications in CO_2_ conversion.

In summary, zeolite membranes have been successfully applied in the field of CO_2_ conversion, including LTA, FAU, CHA, MOR and ZSM-5 membranes. LTA membranes, known for their superior hydrophilicity and small pore size, are suitable for nearly all permeable ZMR applications. Membranes such as H-FAU and H-ZSM-5, which contain acidic sites, can function as both catalytic and separation layers. The balance between hydrophilicity and acidity must be carefully controlled, particularly by adjusting the SAR. Although MOR membranes have been successfully applied in CO_2_ conversion, careful pore size reduction and maintenance of hydrophilicity are required to meet the separation requirements during application.

Other zeolite membranes face limitations in ZMR applications due to intrinsic properties or challenges in fabrication. For example, while SOD membranes exhibit excellent hydrophilicity, their small pore size may compromise permeability. Currently, water-permeable zeolite membranes, leveraging their hydrophilic properties, have been successfully employed for *in situ* water removal in CO_2_-catalyzed reactions. However, H_2_-selective membranes, such as MFI, DDR and CHA membranes, have yet to be successfully applied for *in situ* dehydrogenation in CO_2_-catalyzed reactions, representing a promising direction for future research on e.g. the dehydrogenation of formic acid and the dehydrogenation of methanol for obtaining green hydrogen.

### Challenges and mitigation strategies for zeolite membranes in ZMRs

Although a number of zeolite membranes have been successfully applied in ZMRs for the catalytic conversion of CO_2_ into value-added chemicals, significant challenges remain before this highly innovative strategy can be widely implemented and scaled up for industrial applications. First, ZMRs impose much more stringent requirements on membrane quality than conventional separation processes. In particular, the membranes must simultaneously achieve high separation performance while operating under harsh reaction conditions, including high temperature, high pressure and the presence of reactive species such as water [73]. Second, strongly hydrophilic zeolite membranes are typically associated with high Al content, and the resulting strong electronegativity can lead to intercrystalline defects during membrane growth [50]. In addition, high-Al zeolite membranes generally exhibit lower hydrothermal stability. Finally, challenges related to scalability and reproducibility must be addressed to enable industrial-scale deployment. In the following, these challenges and their corresponding mitigation strategies will be discussed in detail.

#### Fabrication of high-quality zeolite membranes

In contrast to conventional membrane-based separation systems, ZMRs operate under simultaneous high temperature, high pressure and chemically reactive environments, where water, hydrogen, CO_2_ and organic intermediates coexist. Under such conditions, zeolite membranes are required to deliver both high selectivity and high permeability, while maintaining structural integrity over extended periods. This imposes exceptionally strict requirements on membrane quality. Specifically, the membrane must possess minimal defects and well-intergrown crystal boundaries to ensure effective molecular sieving. In addition, a defect-free membrane should also exhibit high permeance to enable efficient reactor operation.

**Figure 4.**
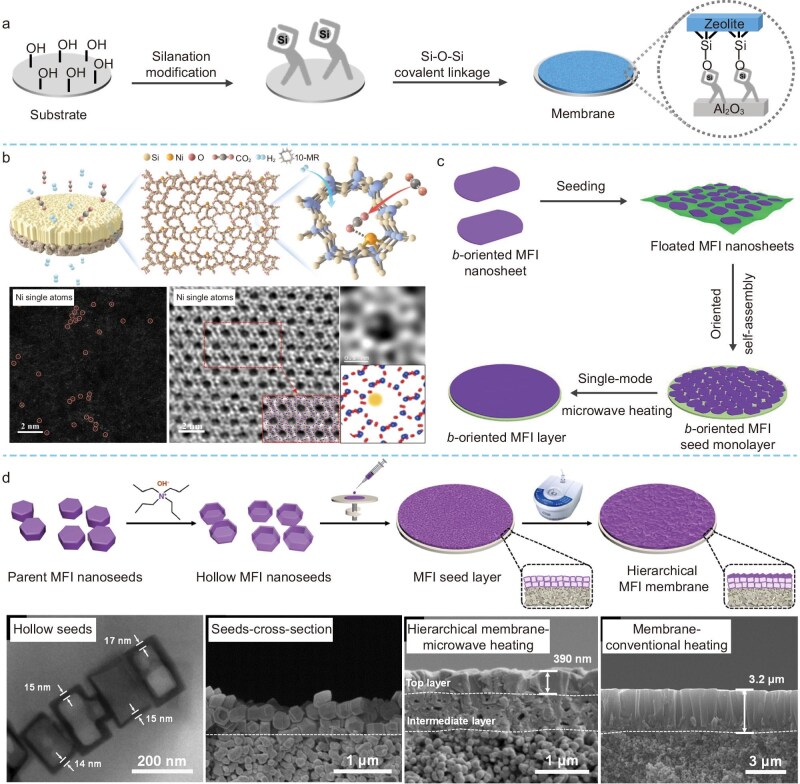
Schematic diagrams for (a) *in situ* synthesis strategy of zeolite membrane and (b) the Ni_1_@S-1 zeolite membrane for single-atom gas separation, along with an aberration-corrected scanning transmission electron microscopy (AC-STEM) image and an integrated differential phase contrast STEM (iDPC-STEM) image revealing the spatial distribution of Ni atoms within the zeolite membrane channels; reproduced with permission from Ref. [81]. Copyright 2025, American Chemical Society. (c) *b*-oriented MFI membrane using *b*-oriented MFI nanosheets; reproduced with permission from Ref. [83]. Copyright 2020, American Association for the Advancement of Science. (d) Schematic of the fabrication of hierarchical MFI membrane via microwave-assisted synthesis strategy; hollow seeds and comparison of MFI membranes prepared with microwave heating and conventional heating; reproduced with permission from Ref. [86]. Copyright 2021, Wiley-VCH GmbH.

The fabrication of defect-free zeolite membranes remains a longstanding challenge, not only for ZMR applications but also for zeolite membranes used in conventional separation processes. This difficulty arises from the inherent nature of zeolite crystal intergrowth, where defects are almost inevitably generated during membrane formation [49,51]. Numerous reviews and studies have already summarized the general synthesis strategies for zeolite membranes [33,74–76]; therefore, this review will not reiterate these aspects. Instead, we focus on representative approaches that are particularly relevant to the preparation of high-quality zeolite membranes for ZMRs. For example, to enhance the interaction between the zeolite membrane and the substrate, as well as to promote intercrystalline growth, Caro *et al.* introduced a novel *in situ* synthesis strategy to fabricate defect-free zeolite membranes for ZMR applications: creation of bridges to the zeolite Si-OH by silanation of the substrate (Fig. 4a). For example, they used 3-aminopropyltriethoxysilane as a covalent linker to build a robust ‘bridge’ between the zeolite membrane and the support [77,78].

Secondary growth strategy is highly regarded for fabricating high-quality zeolite membranes, owing to their ability to deliver precise control over membrane properties and minimize defects [51]. Yu *et al.* [36] designed rational synthesis routes to prepare water-conducting LTA membranes for boosting CO_2_ conversion to CH_3_OH in ZMR. In their process, high-density nanoseeds (50–200 nm) were solidly anchored onto the support by annealing. Yoon *et al.* successfully developed oriented MFI membranes using the gel-free secondary growth technique, in which crystals are aligned in a specific direction, are highly beneficial for reducing intercrystalline defects [79].

Microwave-assisted synthesis strategy has also emerged due to rapid and uniform heating, enabling faster membrane growth while offering precise control over morphology [80]. This approach offers significant advantages in the fabrication of zeolite membranes, including enhanced efficiency, superior membrane quality, reduced energy consumption, scalability and sustainability. Yang *et al.* successfully scaled up the microwave-assisted synthesis of LTA membranes for industrial applications, demonstrating their practical potential in ZMR.

The fundamental role of defect-free zeolite membranes is to ensure high separation selectivity in membrane reactors. In addition to directly reducing intercrystalline defects, the intrinsic tunability of zeolite membranes, such as the SAR, extra-framework cations and pore structure, can be strategically utilized to regulate the interactions between the membrane and permeating molecules. For example, by precisely confining Ni single atoms within the channels of oriented zeolite membranes, CO_2_ molecules can undergo specific chemisorption with Ni sites, whereas H_2_ does not exhibit such interaction (Fig. 4b). This enables the construction of highly efficient chemically selective separation centers, achieving ultrahigh selectivity for H_2_/CO_2_ separation [81].

Due to the harsh operating conditions of ZMR, zeolite membranes are expected to exhibit higher permeation efficiency within limited residence times. To address the challenge of fabricating highly permeable membranes, various strategies have been explored, including controlling crystal orientation, reducing membrane thickness and introducing mesopores to decrease diffusion resistance and accelerate mass transport. For example, Guo *et al.* reported that oriented LTA membranes possess well-aligned grain boundaries that are more favorable for gas permeation [82]. In addition, oriented membranes can optimize the accessibility of target channels, allowing permeating molecules to preferentially diffuse along the most favorable pore directions, thereby significantly reducing diffusion resistance [79].

Ultrathin zeolite membranes, owing to their substantially reduced thickness, can effectively shorten molecular diffusion pathways. In particular, the use of two-dimensional zeolite nanosheets as seeds has brought transformative advances in the fabrication of ultrathin membranes. For instance, Liu *et al.* prepared a sub-100 nm-thick *b*-oriented MFI membrane originated from *b*-oriented MFI nanosheets (Fig. 4c) [83]. In contrast, for small-pore hydrophilic membranes, the development of 2D nanosheets is restricted by intrinsic framework characteristics. Nevertheless, Chao *et al.* innovatively utilized the structural similarity of double six-membered rings shared by different zeolites to develop flaky MCM-22 seeds for synthesizing SOD membranes, resulting in submicron SOD membranes with enhanced water permeation [84]. For LTA membranes, Guo *et al.* reported the fabrication of submicron-thick LTA membranes via a self-limiting growth approach driven by the synergistic interaction between a highly reactive gel and nanosized seeds (50–150 nm) [85].

Furthermore, introducing additional mesopores or macropores into zeolite membranes is an effective strategy to enhance permeability. For example, Liu *et al.* fabricated hierarchical MFI membranes with hollow-structured seeds under microwave-assisted conditions, achieving improved n-butane permeation (Fig. 4d) [86]. Zhang *et al.* prepared hierarchical MFI membranes derived from multidimensionally assembled (2D@0D) seeds [87]. Wang *et al.* [88] introduced macropores into an LTA membrane, significantly enhancing flux for ethanol/water mixtures. These strategies provide feasible pathways for the preparation of highly permeable zeolite membranes and enable more efficient *in situ* removal of by-products in ZMR systems.

#### Conflict between hydrophilicity and strong electronegativity in high-Al-content zeolite membranes

Efficient removal of water is a central requirement in ZMRs for CO_2_ catalytic conversion, particularly for reactions such as methanol synthesis. To achieve strong water affinity, zeolite membranes are typically designed with low SARs. However, the introduction of high Al content also brings inherent challenges to membrane fabrication. The increased framework electronegativity makes intergrowth between crystals more difficult, leading to the formation of intercrystalline defects [50]. This creates a fundamental trade-off: while higher aluminum content improves water adsorption and selectivity, it simultaneously increases the difficulty of achieving high-quality, defect-free membranes.

Resolving this conflict requires precise control over the seed layer design and crystallization process during the fabrication of hydrophilic membranes, to minimize defect formation as much as possible. For example, as mentioned above, chemical bonding strategies can be employed to enhance the interaction between zeolite crystals and the substrate [77], while also providing abundant nucleation sites for intercrystalline growth. The use of small-sized seeds with high-density distribution can further promote crystal intergrowth [85]. In addition, selecting oriented membranes with orderly crystal alignment as the synthesis target helps reduce non-selective transport pathways. Moreover, advanced techniques such as microwave-assisted heating can be utilized to finely regulate the kinetic balance between nucleation and crystal growth, leading to more uniform grain sizes and reduced intrinsic defects between crystals.

#### Conflict between hydrophilicity and hydrothermal stability in high-Al-content zeolite membranes

In addition to strong electronegativity, low-silica hydrophilic zeolite membranes with high Al content also face challenges related to structural instability. During CO_2_ hydrogenation, water is continuously generated at elevated temperatures. Under such high-temperature and high-pressure hydrothermal conditions, prolonged operation can induce dealumination in low-silica zeolite membranes, ultimately leading to framework collapse and alteration of the pore structure [30]. Although increasing the SAR can significantly enhance hydrothermal stability, it simultaneously reduces membrane hydrophilicity, thereby weakening its ability for selective water removal. This highlights a critical design challenge, namely how to achieve a balance between hydrothermal stability and separation functionality.

One effective strategy is to develop zeolite membranes with high permeability, thereby improving water removal efficiency and minimizing the exposure time of the membrane to harsh hydrothermal environments. In addition, to enhance structural stability, composite membrane designs can be adopted by integrating hydrophilic low-silica zeolites with more stable zeolite frameworks. This approach preserves the hydrophilicity of low-Si/Al membranes while improving overall structural robustness. Such designs can be realized through interzeolite conversion strategies, which exploit the structural similarities between different zeolite frameworks. For example, Zou *et al.* [89] developed an interzeolite conversion approach to prepare a NaA membrane on NaX membrane. The structural resemblance between LTA and FAU zeolites, both of which share the same SOD building unit, served as the driving force for this conversion. This interzeolite conversion strategy provides a feasible pathway for enhancing the overall stability of zeolite membranes.

#### Scalability for industrial applications

The industrial application of CO_2_ hydrogenation to produce methanol has been successfully achieved. From an application perspective, the transition of ZMR technology from laboratory research to industrial implementation depends heavily on the ability to fabricate zeolite membranes with high reproducibility and scalability. In large-scale fabrication, maintaining uniform membrane thickness, consistent seed distribution and defect-free intergrowth across extended areas or complex geometries (e.g. tubular or multichannel configurations) remains a significant challenge [33,49]. In addition, conventional hydrothermal synthesis methods are often associated with high energy consumption, long processing times and significant solvent usage, which hinder economic feasibility. Moreover, the fabrication cost of zeolite membranes is inherently high, which further increases the overall cost of ZMR systems and necessitates the development of membranes with high permeation efficiency. Therefore, an ideal preparation method for ZMR applications must integrate functionality, cost-effectiveness, environmental sustainability, reproducibility and industrialization potential.

Currently, LTA membranes commonly used in water-permeable membrane reactors have been industrialized through secondary growth and microwave-assisted synthesis strategies. Tubular LTA membrane reactors with lengths up to 210 mm have been successfully applied in CO_2_ hydrogenation to methanol [36]. In addition, multichannel hollow fiber CHA membrane reactors fabricated via secondary growth have also been successfully applied to CO_2_ conversion, demonstrating the strong potential of ZMRs for large-scale applications [60]. However, considering economic and environmental factors, emerging strategies such as gel-free secondary growth [79], solvent-free synthesis [90] and room-temperature fabrication [91] should be further integrated into current large-area zeolite membrane preparation approaches.

**Figure 5.**
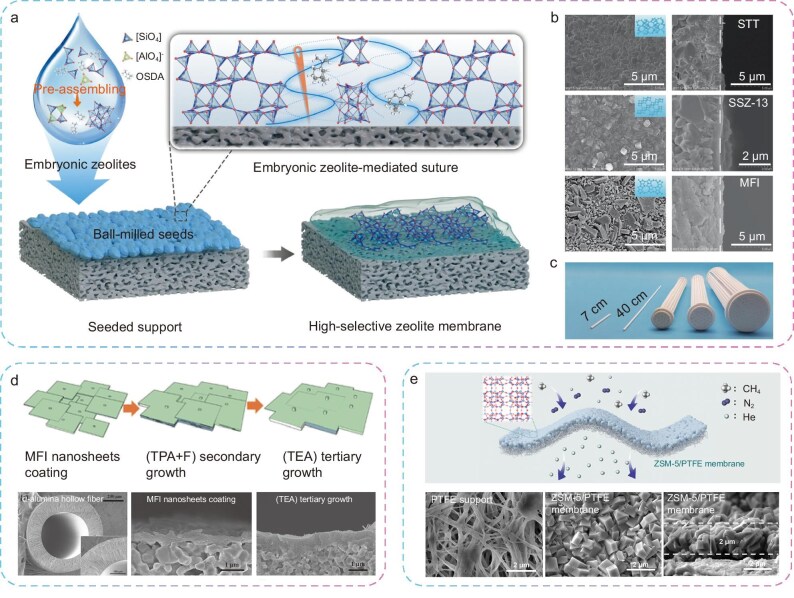
(a) Illustration of embryonic zeolite-mediated suture (EZMS) strategy. (b) STT, SSZ-13 (belong to Chabazite (CHA)) and MFI membranes synthesized via EZMS strategy. (c) Large-area zeolite membrane synthesized via EZMS strategy; reproduced with permission from Ref. [94], Copyright 2026, Springer Nature. (d) An MFI membrane fabricated from two-dimensional nanosheets on an alumina hollow fiber substrate; reproduced with permission from Ref. [95]. Copyright 2019, Wiley-VCH GmbH. (e) Flexible ZSM-5 membrane synthesized on PTFE support; reproduced with permission from Ref. [96]. Copyright 2024, Wiley-VCH GmbH.

In addition, the preparation of commercial hollow fiber LTA membrane assemblies is well-established. And a series of large-area CHA and DDR hollow fiber membranes have also been successively developed [92,93]. Recently, a novel strategy was developed by Nanjing Tech University [94] (Fig. 5a), in which pre-assembled short-range ordered zeolite embryos were used as reactive intermediates. Through condensation reactions, ball-milled seed particles were seamlessly assembled into continuous and dense zeolite membranes, with membrane thickness precisely controlled at the level of the initial seed layer. This approach has been successfully demonstrated for STT, CHA and MFI topologies (Fig. 5b). Notably, it enabled the scalable fabrication of a single 40-cm long hollow fiber membrane as well as a membrane module consisting of 102 fibers with an area of 0.5 m^2^ (Fig. 5c). All membrane modules exhibited CO_2_/CH_4_ selectivity exceeding 100 and maintained long-term stability for over 220 days. MFI membranes fabricated from 2D nanosheet on ceramic hollow fiber [95] demonstrate the great potential for large-area fabrication of such high-permeability membranes (Fig. 5d). In addition, Zou *et al.* successfully prepared ZSM-5 membranes on polytetrafluoroethylene (PTFE) substrates, providing a feasible strategy to reduce the fabrication cost of zeolite membranes, which is largely dominated by the expensive alumina supports [96] (Fig. 5e). These technological advances are expected to open new opportunities for the development of ZMRs.

Overall, the challenges associated with zeolite membranes in ZMRs originate from the need to simultaneously satisfy multiple, and often competing, requirements: high selectivity, high permeability, structural robustness, hydrothermal stability and industrial feasibility. These challenges are intrinsically interconnected, forming a complex design landscape. Addressing these issues requires a shift toward integrated design strategies that combine precise control of membrane composition, microstructure and fabrication processes with reactor-level considerations. Only through such a holistic approach can zeolite membranes be fully optimized to meet the demands of practical CO_2_ catalytic conversion in membrane reactors.

### Design strategies of ZMRs

**Figure 6.**
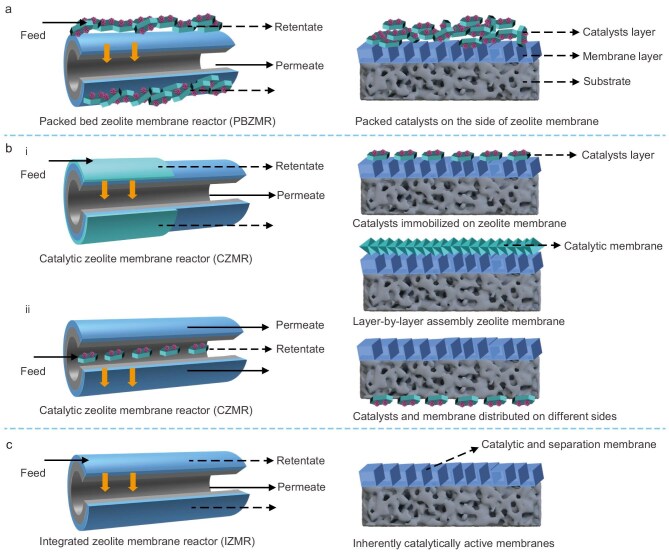
Classification of ZMR configurations.

The coupling strategy between catalysts and zeolite membranes plays a decisive role in determining not only the catalytic performance but also the long-term stability of ZMRs. Beyond simple structural differences, each configuration fundamentally alters mass transfer pathways, catalysts loading, interfacial interactions and local reaction environments, thereby influencing catalyst utilization efficiency and deactivation behavior [30]. In the context of CO_2_ catalytic conversion, these effects become particularly critical due to the sensitivity of catalysts to water, temperature and intermediate species. Several combinations of catalysts and zeolite membranes have been explored, as summarized below:

(1) Packed-bed zeolite membrane reactor: In this case, catalysts are directly packed on the sides of the membrane forming the packed-bed zeolite membrane reactor (PBZMR). The reaction occurs in the catalyst layer, while the product is separated through the membrane (Fig. 6a). For example, Gu *et al.* developed MFI-type membrane reactors with a catalytic packed bed on the tube side of the membrane for meta-xylene isomerization [97]. This configuration allows for a high catalyst loading and shows promise for large-scale application of ZMRs. Similarly, Yu *et al.* achieved a catalyst loading of 2.8 g on a 210-mm length LTA membrane [36]. Huang *et al.* fabricated a 19-channel monolithic CHA membrane packed with catalyst [60]. The development from single-tube membrane reactors to multichannel modular membrane reactors demonstrates the successful transition from the laboratory scale to the pilot-scale stage, laying a solid foundation for future industrial-scale applications.

However, the spatial separation between the catalysts and membrane surface introduces intrinsic limitations in reaction–separation coupling. From a performance perspective, the transport of products from the catalyst layer to the membrane relies on diffusion across the packed bed. This results in delayed product removal, particularly for strongly adsorbing species such as water in CO_2_ hydrogenation. The accumulation of such species can shift reaction equilibrium unfavorably and promote side reactions, thereby reducing catalytic efficiency. More importantly, this weak coupling significantly impacts stability. The insufficient removal of water and intermediates can accelerate metal sintering and catalyst deactivation. Therefore, although PBZMRs are structurally simple and scalable, their stability is often limited by inefficient mass transfer and weak interfacial interaction.

(2) Catalytic zeolite membrane reactor: Catalysts can be directly loaded onto the membrane surface or integrated with it to construct catalytic zeolite membrane reactors (CZMRs) (Fig. 6b), leading to a significantly enhanced interfacial coupling between reaction and separation processes. This strong coupling configuration shortens the diffusion distance between catalytic sites and membrane pores, enabling near-immediate removal of by-products. In CO_2_ hydrogenation, the layered geometry in CZMRs allows water to be rapidly removed from the reaction zone. Moreover, since all reactant molecules are forced to pass through the catalytic layer, more effective contact between reactants and active sites can be achieved. As a result, Huang *et al.* reported that CZMRs exhibited 100% methanol selectivity in CO_2_ hydrogenation to methanol, along with a relatively high CO_2_ conversion [56].

However, a major limitation of CZMRs is the relatively low catalyst loading. Conventional impregnation methods still face challenges, such as overcoming capillary forces within membrane pores. In addition, the catalytic layer is prone to detachment, resulting in poor mechanical stability of the membrane reactor. Additionally, the close proximity between the catalyst and membrane increases the likelihood of membrane pore blockage and surface fouling, especially under long-term operation. To address these issues, researchers have explored various strategies, including surface modification of membranes and alternative loading methods such as deposition and flow-through techniques [24], to enhance the loading of active components and strengthen the interaction between the catalyst and the membrane.

An alternative approach to preparing CZMRs involves layer-by-layer assembly of zeolite membranes via hydrothermal growth (Fig. 6b). This method creates a sandwich-like configuration with an upper catalytic layer, a middle separation layer and a lower support layer. For example, Huang *et al.* reported methanol dehydrogenation to DME using double membrane ZMRs constructed using H-FAU and LTA membranes as the catalysis and separation layers, respectively [98]. This design enhances interlayer interactions, resulting in a hierarchical pore structure, distinct hydrophilic and hydrophobic properties and tailored acidity, all of which benefit the reaction. However, the double-layer membrane design increases the overall thickness, reducing membrane permeability and potentially impacting performance. Thus, the stability of CZMRs is governed by a delicate balance between interfacial integration and transport resistance.

In summary, this type of membrane reactor features a highly designable structure and enables the integration of reaction and separation. However, during long-term operations, issues such as pore blockage and flux decline may arise. Therefore, such ZMRs should first focus on optimizing loading strategies and interfacial engineering, while further advancing toward modularization.

(3) Integrated zeolite membrane reactor: In integrated zeolite membrane reactors (IZMRs), zeolite membrane simultaneously functions as both the catalytic medium and the separation layer (Fig. 6c), representing the highest degree of reaction–separation coupling. This intrinsic integration eliminates interfacial resistance and ensures that reactions occur directly within or in close proximity to the membrane pores. Tarditi *et al.* reported a Ba^2+^-exchanged ZSM-5 membrane reactor employed for xylene isomerization [99]. Gu *et al.* synthesized a pure-silica MFI membrane with an Al-MFI intermediate layer. The incorporated Al, orienting from the support and subjected to H^+^-exchange, served as a catalytic medium for xylene isomerization, while the pure-silica MFI layer facilitated product separation [100].

Such a configuration offers unique advantages in terms of maximized coupling efficiency and minimized diffusion limitations, which can significantly enhance catalytic performance. Moreover, the absence of a distinct catalyst layer reduces issues related to catalyst detachment or poor interfacial contact, potentially improving structural stability. However, this type of membrane reactor is inherently limited to reactions that rely on the intrinsic acidity of the zeolite as active sites and on separation processes governed by specific pore channels.

Currently, most CO_2_ catalytic conversion applications using ZMRs fall under the PBZMR and CZMR configurations due to their versatility. For PBZMRs, improving the interaction forces between the two phases remains a priority. For CZMRs, increasing the catalyst loading on the membrane is critical to enhancing catalytic efficiency. Future developments should focus on designing hybrid coupling strategies that combine high catalyst loading with strong interfacial integration. Such advances are expected to significantly enhance both the catalytic efficiency and long-term stability of ZMRs for CO_2_ conversion applications.

### Progress in ZMRs for CO_2_ catalytic conversion

This section provides a comprehensive overview of recent progress in ZMRs for CO_2_ catalytic conversion. It begins with a systematic comparison of the performance of ZMRs and FBRs across representative reaction pathways, including CO_2_ hydrogenation to methanol, conversion to DME and carbonate synthesis. Table 2 summarizes the performance of ZMRs and traditional FBRs in CO_2_ conversion reactions. Subsequently, the stability of ZMRs is discussed, with a focus on the durability of zeolite membranes and their impact on long-term reactor operation. Finally, recent advances in process simulation are summarized, emphasizing the role of modeling in understanding reaction–separation coupling.

**Table 2.** ZMRs for CO_2_ catalytic conversion.

Chemicals	Reactor type	Temperature/pressure	Conversion/CO_2_	Selectivity	Membrane	Ref.
Methanol	ZMR	210°C	17%		LTA	[61]
	FBR		6%			
Methanol	ZMR	220°C, 3.5 MPa	57.2%	70%	NaA	[36]
	FBR		23%	60%		
Methanol	ZMR	260°C, 3 MPa	36.1%	100%	LTA	[56]
	FBR		21.9%	67.3%		
Methanol	ZMR	260°C, 3 MPa	49.1%	90.2%	LTA	[101]
	FBR		26.2%	50.6%		
Methanol	ZMR	230°C, 0.5 MPa	12%	1%	LTA	[35]
Methanol	ZMR	250°C, 3 MPa	35.2%	96.7%	Cu-MOR	[54]
	FBR		15.4%	75.6%		
Methanol	ZMR	275°C, 3 MPa	37.6%	93.4%	CHA	[60]
	FBR		30.3%	92.0%		
DME	ZMR	280°C, 3.25 MPa	41.1%	100%	H-ZSM-5	[46]
	FBR		24.9%	53.7%		
DME	ZMR	270°C, 5 MPa	73%	35%	SOD	[102]
	FBR		30%	16%		
DME	ZMR	275°C, 3 MPa	70%	60%	SOD	[103]
DME	ZMR	300°C, 2.8 MPa	22.8%	33.7%	NaA	[104]
	FBR		8.71%	21.4%		
DME	ZMR	275°C, 4 MPa	20%	60%	LTA	[105]
DME	ZMR	240°C, 4 MPa	52%	38%	CZM	[106]
	FBR		29%	58%		
DME	ZMR	250°C, 3.5 MPa	73.4%	74.3%	LTA	[107]

CZM, carbon zeolite membrane.

#### Performance of different kinds of ZMRs for CO_2_ conversion

*CO_2_ hydrogenation to methanol.* Methanol, as a renewable energy carrier, serves as a valuable feedstock, and CO_2_ hydrogenation to methanol has attracted significant attention for its potential to reduce CO_2_ emissions [108]. This reaction is exothermic (Eq. 1), with product distribution influenced by operating conditions such as temperature, pressure and reactant ratio. Generally, the reaction competes with the reverse water-gas shift (RWGS) reaction (Eq. 2) [109].


(1) \begin{eqnarray*}
&&{\mathrm{C}}{{\mathrm{O}}}_2 + 3{{\mathrm{H}}}_2 \Leftrightarrow {\mathrm{C}}{{\mathrm{H}}}_3{\mathrm{OH}} + {{\mathrm{H}}}_2{\mathrm{O}},\\
&&\qquad\Delta {{\mathrm{H}}}_{298\,{\mathrm{K}}} = -\, 49.5\,{\mathrm{kJ}}\,{\mathrm{mo}}{{\mathrm{l}}}^{ - 1};
\end{eqnarray*}

(2) \begin{eqnarray*}
&&{\mathrm{C}}{{\mathrm{O}}}_2 + {{\mathrm{H}}}_2 \Leftrightarrow {\mathrm{CO}} + {{\mathrm{H}}}_2{\mathrm{O}},\\
&&\qquad \Delta {{\mathrm{H}}}_{298\,{\mathrm{K}}} = +\, 41.2\,{\mathrm{kJ}}\,{\mathrm{mo}}{{\mathrm{l}}}^{ - 1}.
\end{eqnarray*}

Currently, Cu-based catalysts are the most widely used for this reaction and dominate industrial applications [110]. However, these catalysts face significant challenges due to the competing RWGS reaction and water-induced deactivation, leading to inadequate activity [111]. In particular, the reaction is limited by the thermodynamic equilibrium, making it difficult to achieve both high CO_2_ conversion and CH_3_OH yield [112]. While high temperatures enhance catalyst activation, low temperatures are thermodynamically favorable for the reaction, creating a delicate balance between kinetic and thermodynamic limitations. The selective removal of water using a membrane reactor can promote the forward reaction and inhibit catalyst deactivation, thereby increasing CO_2_ conversion and addressing these challenges [113]. Hydrophilic ZMRs have been effectively used in this reaction, achieving significant higher CO_2_ conversion or CH_3_OH yield than traditional FBRs [36,109].

Tavolaro *et al.* [61] were the first to report the use of an LTA membrane loaded with Cu/ZnO/Al_2_O_3_ catalysts as a membrane reactor for CO_2_ hydrogenation to methanol. The LTA membrane was prepared on the inner tubular surface of the substrate and packed with catalysts. Such ZMR achieved a CO_2_ conversion of ∼17%, nearly triple that of the traditional reactor module, highlighting its improved catalytic performance. However, the membrane’s permeability followed the Knudsen mechanism with very low selectivity, resulting in similar CO_2_ conversion between such ZMR and the traditional reactor at high reaction temperatures.

**Figure 7.**
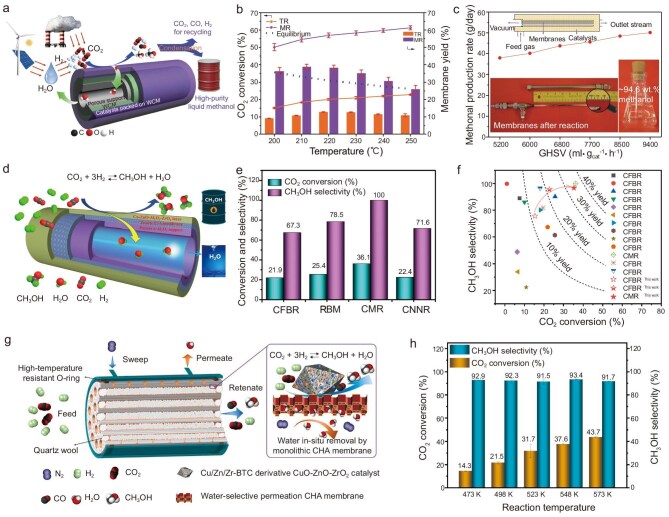
(a) Schematic of WCM-incorporated dehydration ZMR for high-purity methanol direct synthesis from renewable resources. (b) Catalytic CO_2_ conversion and methanol yield obtained in the traditional reactor and in the membrane reactor as a function of temperature at 35 bar and feed (CO_2_/H_2_ = 1/3). (c) High-purity methanol direct synthesis in the membrane reactor at different feed GHSV at 220°C and 35 bar with three hollow fiber membranes 210 nm in length. Reproduced with permission from Ref. [36]. Copyright 2020, American Association for the Advancement of Science. (d) CO_2_ hydrogenation to methanol through *in situ* and continuous removal of water in a reaction–separation bifunctional LTA@Cu-ZnO-Al_2_O_3_-ZrO_2_ CMR. (e) Catalytic performance of the CMR in the CO_2_ hydrogenation to methanol in comparison with the CFBR, PBMR and catalytic non-permselective membrane reactor. Reproduced with permission from Ref. [56]. Copyright 2021, Wiley-VCH GmbH. (f) Comparison of CO_2_ conversion and methanol selectivity achieved by the Cu-MOR CMR with those reported for various reactor configurations in CO_2_ hydrogenation to methanol. Reproduced with permission from Ref. [54]. Copyright 2025, Elsevier. (g) Diagram of methanol synthesis from CO_2_ in a catalyst/CHA multichannel PBMR. (h) Catalytic performances of CO_2_ hydrogenation to methanol catalytic in CHA membrane reactor as a function of reaction temperatures at 3.0 MPa. Reproduced with permission from Ref. [60]. Copyright 2025, Elsevier.

Yu *et al.* [36] later reported a packed ZMR with unprecedented CO_2_ conversion (Fig. 7a). A defect-free NaA membrane, created via thermal annealing approach, exhibited high H_2_O/gas selectivity, with the Cu-ZnO-Al_2_O_3_-ZrO_2_ catalyst packed on both sides of the membrane. Efficient *in situ* H_2_O removal through the WCM substantially enhanced CO_2_ conversion and CH_3_OH yield in the ZMR (i.e. 61% and 809 mg g_cat_^−1^ h^−1^, respectively) compared to the traditional reactor (i.e. 23% and 339 mg g_cat_^−1^ h^−1^, respectively) (Fig. 7b). The ZMR demonstrated stable operation for over 100 h, producing high-purity CH_3_OH and showing promise for large-scale applications (Fig. 7c). Further, they reported on the methanol to liquified petroleum gas (LPG) over bifunctional Cu/Zn/Zr on Al_2_O_3_ (CZZA)/Pd-β-zeolite catalyst, ultimately coupling ZMR to realize LPG yield of 51.7% and CO_2_ conversion of 72.1% [114]. Huang *et al.* [56] reported a bifunctional ZMR for CO_2_ conversion with highly efficient methanol selectivity (i.e. 100%) (Fig. 7d and e). The Cu-ZnO-Al_2_O_3_-ZrO_2_ catalyst was loaded on the surface of the LTA membrane. Due to the repaid *in situ* removal of water, the thermodynamic equilibrium limitation was effectively overcome, resulting in improved CO_2_ conversion (36.1%) and methanol selectivity (100%), significantly outperforming the catalytic fixed-bed reactor (CFBR), which achieved a CO_2_ conversion of 21.9% and methanol selectivity of 67.3% (Fig. 7e). The high temperatures cause the aggregation of Cu^0^ particles, resulting in lower activity and decreased CO_2_ conversion. While the endothermic RWGS reaction (Eq. 3) is favored at high temperatures, the ZMR maintained 100% methanol selectivity even at 260°C, as the reverse reaction and catalyst deactivation were effectively prevented due to *in situ* water removal. Additionally, the water removal minimized catalyst deactivation, ensuring stable catalytic activity for over 90 h.

In addition, they found that the MOR ZMR, which exhibits higher stability, showed superior performance for CO_2_ hydrogenation. Using a CuO-ZnO@Cu-MOR catalytic membrane reactor (CMR), a CO_2_ conversion of 35.2% and a methanol selectivity of 96.7% were achieved (Fig. 7f), and the system remained stable for 200 h at 250°C and 3.0 MPa. In this case, the Cu-MOR zeolite membrane not only ensured the hydrophilicity of the separation layer but also possessed smaller pore sizes compared with the MOR membrane [54].

Furthermore, to further enhance the industrial applicability of CO_2_ catalytic conversion in ZMRs, the hydrogenation catalyst CuO-ZnO-ZrO_2_ was packed into a 19-channel monolithic CHA zeolite membrane (Fig. 7g). This system achieved a high CO_2_ conversion of 37.6% and a methanol selectivity of 93.4%, and exhibited stable operation at 275°C and 3.0 MPa for 200 h (Fig. 7h) [60].

Membrane reactors have demonstrated great potential for CO_2_ hydrogenation to methanol by effectively overcoming the limitations of thermodynamic equilibrium and improving product selectivity. Through the integration of catalytic and separation functions, these systems enable the *in situ* removal of by-products such as water, thereby promoting forward reactions and enhancing methanol yields. Various zeolite membranes, including LTA, CHA and MOR, have been successfully applied in ZMRs, exhibiting excellent stability and tunable hydrophilicity, which are critical for efficient CO_2_ conversion. Moreover, the successful fabrication of large-area and multichannel membrane modules marks a significant step toward pilot-scale and industrial applications. Ultimately, the integration of ZMRs into industrial CO_2_ utilization processes could provide an efficient and sustainable route for methanol production, contributing to carbon neutrality and the circular carbon economy.

*CO_2_ conversion to DME*. DME is attracting significant attention as a promising alternative fuel due to its high cetane number and low auto-ignition temperature [115]. Moreover, DME serves as a vital feedstock for the production of light olefins, methyl acetate and other valuable chemicals [116]. Two primary routes have been proposed for converting CO_2_ into DME [117]: (i) the conversion of CO_2_ to CH_3_OH followed by CH_3_OH dehydration to DME (Eq. 3), and (ii) the integration of these two steps into a single process using a bifunctional catalyst (Eq. 4). The CH_3_OH dehydration step is particularly advantageous as it enhances CO_2_ conversion while offering significant economic and thermodynamic advantages.


(3) \begin{eqnarray*}
&& 2{\mathrm{C}}{{\mathrm{H}}}_3{\mathrm{OH}} \Leftrightarrow {\mathrm{C}}{{\mathrm{H}}}_3{\mathrm{OC}}{{\mathrm{H}}}_3 + {{\mathrm{H}}}_2{\mathrm{O}},\\
&&\qquad \Delta {{\mathrm{H}}}_{298\,{\mathrm{K}}} = -\, 23.4\,{\mathrm{kJ}}\,{\mathrm{mo}}{{\mathrm{l}}}^{ - 1};
\end{eqnarray*}

(4) \begin{eqnarray*}
&& 2{\mathrm{C}}{{\mathrm{O}}}_2 + 6{{\mathrm{H}}}_2 \Leftrightarrow {\mathrm{C}}{{\mathrm{H}}}_3{\mathrm{OC}}{{\mathrm{H}}}_3 + 3{{\mathrm{H}}}_2{\mathrm{O}},\\
&&\qquad \Delta {{\mathrm{H}}}_{298\,{\mathrm{K}}xst} = -\, 122.4\,{\mathrm{kJ}}\,{\mathrm{mo}}{{\mathrm{l}}}^{ - 1}.
\end{eqnarray*}

A bifunctional catalyst must possess both CO_2_ hydrogenation activity and sufficient acidity required for CH_3_OH dehydration [66]. The formation of by-product water has a significant impact on the activity and stability of the catalyst. In direct synthesis routes, physical and chemical dehydration may occur i.e. zeolite membranes not only provide acidic sites to facilitate the methanol dehydration process but also serve as separation channels for by-product water removal. More importantly, zeolite membranes exhibit chemical and thermal stability under reaction conditions ranging from 200°C to 300°C and 30–70 bars. Combining these membranes with catalyst for methanol synthesis is a promising approach for the direct conversion of CO_2_ to DME.

**Figure 8.**
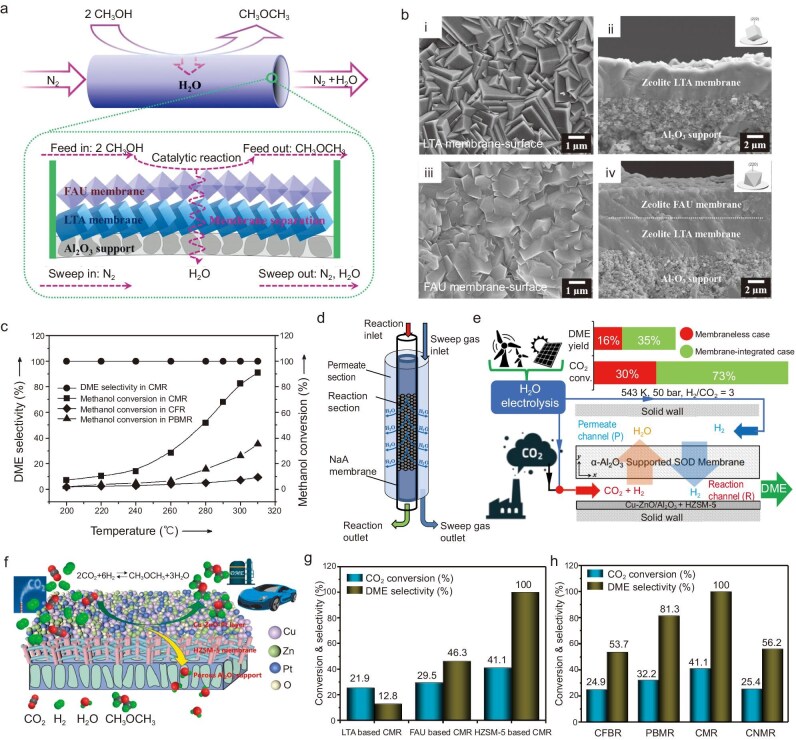
(a) Schematic of the bifunctional CMR based on FAU-LTA double-layer membrane for methanol dehydration to DME. (b) Top view and SEM cross-section images of the LTA membrane and FAU-LTA double-layer membrane. (c) DME selectivity in the CMR and conversion of methanol into DME as a function of the reaction temperature in the different reactors. Reproduced with permission from Ref. [98]. Copyright 2016, Wiley-VCH GmbH. (d) Distribution of PBZMR for CO_2_ hydrogenation to DME. Reproduced with permission from Ref. [105]. Copyright 2021, Elsevier. (e) DME synthesis via CO_2_ hydrogenation in SOD ZMR. Reproduced with permission from Ref. [102]. Copyright 2022, American Chemical Society. (f) ZMR for DME for CO_2_ hydrogenation. (g) Catalytic performances of CO_2_ hydrogenation to DME in different zeolites based CMRs. (h) Catalytic performances of CO_2_ hydrogenation to DME in different reactors. Reproduced with permission from Ref. [46]. Copyright 2022, Elsevier.

Huang *et al.* [98] reported a sandwich structure in which an H-FAU zeolite membrane acts as the catalyst for methanol dehydration to DME, while the underlying LTA zeolite membrane serves as a separation layer for H_2_O/gas (Fig. 8a and b). By enabling the selective and continuous removal of by-products, this design prevented equipment failure and catalyst deactivation. The ZMR achieved a CH_3_OH conversion rate 90.9% higher than that of the FBR (9.5%), with DME selectivity effectively reached 100% (Fig. 8c). Moreover, the system demonstrated excellent stability, maintaining consistent performance for over 60 h.

In the field of direct hydrogenation of CO_2_, Ateka *et al.* [105] conducted a theoretical study on the production of DME in a packed-bed NaA ZMR. The study utilized a CuO-ZnO-MnO/SAPO-18 catalyst to achieve successive production of methanol and DME, with water, the final by-product, removed through the membrane layer (Fig. 8d). Subsequently, Avci *et al.* [102] achieved a CO_2_ conversion of 30% and DME selectivity of 73% on Cu-ZnO/Al_2_O_3_/H-ZSM-5-SOD membrane reactor, compared to a CO_2_ conversion of 16% and DME selectivity of 35% in the membrane-less case (Fig. 8e). Notably, Huang *et al.* further reported a H-ZSM-5 membrane reactor loaded with a Cu-ZnO-Pt catalyst for the direct CO_2_ hydrogenation to DME [46] (Fig. 8f). H-ZSM-5 not only provides acidity for methanol dehydration to DME, but also acts as a separation layer for physical removal of by-product water. Obviously, H-ZSM-5 membrane reactor showed higher catalytic activity than LTA and FAU membranes due to its appropriate acidity, which is required for methanol dehydration to DME (Fig. 8g). The continuous removal of by-product water effectively overcame the thermodynamic equilibrium limitation, significantly enhancing CO_2_ conversion (from 24.9% in the CFBR to 41.1% in the ZMR) and improving DME selectivity (from 53.7% in the CFBR to 100% in the ZMR) (Fig. 8h).

ZMR provides a promising and energy-efficient pathway for the direct synthesis of DME from CO_2_. The precise control of acidity and pore structure in zeolite membranes is essential to balance methanol dehydration and water permeation. Future efforts should focus on the development of large-area, multichannel and modular membrane reactor designs that can facilitate scale-up from laboratory to pilot applications. With continued innovations in membrane materials, reactor architecture and catalyst-membrane integration, ZMR technology is expected to play a vital role in the sustainable utilization of carbon resources and the future clean fuel industry.

*CO_2_ conversion to carbonate.* DMC and diethyl carbonate (DEC) have attracted increasing attention in the chemical and automotive fuel industries due to their high oxygen content and versatile applications [118]. These carbonates can serve as environmentally benign fuel additives to improve gasoline combustion efficiency, and are also widely used in pharmaceutical synthesis and as electrolyte components in lithium-ion batteries [118]. Among the various synthetic routes, the direct conversion of CO_2_ with methanol (or ethanol) to produce carbonates is particularly attractive because the feedstocks are inexpensive and non-toxic [118]. However, this reaction is strongly limited by unfavorable thermodynamics, leading to extremely low equilibrium yields (Eq. 5) [19]. Moreover, the generation of water as a by-product shifts the equilibrium backward and accelerates catalyst deactivation. Therefore, membrane reactors capable of selectively removing water are considered a promising strategy to overcome these limitations, and several recent reviews have highlighted the potential of membrane-assisted dehydration for this reaction system.


(5) \begin{eqnarray*}
&&{\mathrm{C}}{{\mathrm{O}}}_2 + 2{\mathrm{C}}{{\mathrm{H}}}_3{\mathrm{OH}} \Leftrightarrow ({\mathrm{C}}{{\mathrm{H}}}_3{\mathrm{O}})2{\mathrm{CO}}+ {{\mathrm{H}}}_2{\mathrm{O}},\\
&&\qquad \Delta {{\mathrm{G}}}_{298\,{\mathrm{K}}} = 26.2\,{\mathrm{kJ}}\,{\mathrm{mo}}{{\mathrm{l}}}^{ - 1}.
\end{eqnarray*}

Representative experimental studies have demonstrated the feasibility of this concept. For instance, Wohlrab and co-workers investigated CMRs equipped with various inorganic porous membranes, including MFI membranes (Si/Al 57 and Si/Al 270), NaA zeolite membrane and carbon zeolite membrane, for the continuous esterification of CO_2_ with ethanol to produce DEC. The results revealed that enhanced water removal significantly improved DEC formation, and the use of hydrophilic zeolite membranes increased the productivity by up to 43% compared with conventional FBR under identical conditions [119]. Similarly, Li *et al.* developed a CMR combining Cu-KF/MgSiO catalysts with different membranes, including mesoporous silica, polyimide–silica hybrid membrane and polyimide–titania hybrid membranes, for the direct synthesis of DMC from CO_2_ and methanol. In the presence of membranes, the DMC yield was consistently higher than that obtained in the absence of membranes, with the polyimide–silica hybrid membrane achieving the highest yield of 8.8% [120].

Despite these encouraging results, the application of membrane reactors for CO_2_-to-carbonate synthesis remains at an early stage. Existing studies nevertheless confirm that *in situ* water removal via selective membranes can effectively shift the reaction equilibrium and enhance carbonate formation. It should be noted that this reaction also faces intrinsic kinetic challenges. Compared with many other CO_2_ conversion reactions, the coupling between CO_2_ and alcohols involves a relatively high activation barrier [121], making the development of efficient catalysts particularly challenging. In addition, the highly unfavorable thermodynamics of carbonate formation (nearly non-spontaneous under ambient conditions) further emphasize the importance of equilibrium-shifting strategies such as membrane-assisted dehydration. Therefore, future progress in this field will rely on the synergistic development of highly active catalysts and membranes with high water selectivity and permeance.

Overall, the applicability and performance of ZMRs in CO_2_ catalytic conversion strongly depend on the intrinsic thermodynamic and kinetic characteristics of the target reactions, as well as their sensitivity to by-product water. For CO_2_ hydrogenation to methanol, ZMRs are particularly effective due to their ability to selectively remove water, thereby shifting the thermodynamic equilibrium toward methanol formation and suppressing catalyst deactivation. This reaction represents the most mature application of ZMRs, with demonstrated improvements in both CO_2_ conversion and methanol selectivity. In the case of direct CO_2_ hydrogenation to DME, the advantages of ZMRs become even more pronounced, as the process involves consecutive reactions, both of which benefit from continuous water removal. The integration of acidic zeolite membranes further enables simultaneous catalysis and separation, making ZMRs highly suitable for this tandem reaction system. In contrast, the application of ZMRs in carbonate synthesis (e.g. DMC and DEC) is still at an early stage. While membrane-assisted water removal has been shown to enhance carbonate yields, these reactions suffer from severe thermodynamic limitations and high intrinsic energy barriers for CO_2_ activation and C-O coupling, placing greater demands on catalyst design. Future research should focus on reaction-specific optimization of membrane properties, catalyst design and reactor configuration to fully exploit the synergistic effects of reaction–separation coupling in different CO_2_ conversion systems.

#### Stability of zeolite membrane reactor

The stability of the ZMR is a critical factor determining its practical applicability in industrial processes [24]. For ZMR, maintaining long-term catalytic activity and structural integrity under reaction conditions is particularly important, because such CO_2_ utilization reactions are typically conducted under harsh environments involving high temperatures, reactive intermediates and complex feed compositions.

The stability of ZMRs for CO_2_ catalytic conversion is influenced by several factors, including catalyst deactivation, membrane permeability, membrane structural stability and the coupling between reaction and separation processes [20]. For instance, in long-term operation of CO_2_ hydrogenation, high temperatures can lead to the sintering of metal active phases or the migration of active species, while the formation of water will further aggravate this aggregation [7]. Consequently, the water permeance and structural integrity of zeolite membranes directly dictate the performance stability of membrane reactors [20,30]. Zeolite membranes with superior water permeability facilitate the rapid and selective removal of water by-products from the reaction mixture. Furthermore, the coupling configuration of the reaction and separation modules determines the efficiency of by-product removal, which in turn affects the catalyst deactivation rate. While tight coupling between the catalyst and the membrane allows for the immediate evacuation of products from the reaction zone, excessive integration may lead to membrane pore blockage.

Evidently, zeolite membranes play a pivotal role in enhancing the performance of CMRs for CO_2_ conversion [29]. First, the inherent hydrophilicity of these membranes enables rapid *in situ* dehydration, which retards the sintering of metallic particles. Second, the well-defined microporous framework and molecular sieving capabilities of zeolite membranes ensure high selectivity for water transport. Third, their exceptional hydrothermal and chemical stability allows them to maintain structural integrity and separation efficiency under the high-pressure and high-temperature conditions typical of CO_2_ hydrogenation. Ultimately, through the synergistic effect of reaction and separation, ZMRs maintain a more stable environment, thereby alleviating catalyst degradation and extending the operational lifespan of the reactor.

Recent studies have demonstrated the potential of various zeolite membranes for CO_2_ catalytic conversion processes. In particular, water-selective zeolite membranes have shown significant promise in enhancing the stability of catalysts by continuously removing water. For instance, Huang *et al.* [98] reported a sandwich-like H-FAU/Na-LTA membrane reactor for the dehydration of methanol to DME that exhibited sustained stability for over 60 h, whereas the previously reported HY catalyst in a conventional FBR deactivated after only 7 h [122]. Similarly, Yu *et al.* observed negligible deactivation of Cu-ZnO-Al_2_O_3_/H-ZSM-5 catalyst during long-term testing, an effect attributed to the efficient dehydration provided by the LTA zeolite membrane [107]. In a comparative study of Cu-ZnO-Al_2_O_3_-ZrO_2_ catalysts used for CO_2_ hydrogenation to methanol, Huang *et al.* found that under identical reaction conditions, the specific surface area of the catalyst in an FBR plummeted from 263 to 31 m^2^ g^−^^1^. In contrast, the catalyst within the membrane reactor showed only a slight decrease in surface area after 90 h of operation, with its morphology remaining largely intact [56]. Furthermore, post-reaction characterization of Cu-ZnO-based catalysts revealed significant grain agglomeration and sintering, along with structural damage in FBR. However, in the membrane reactor, the Cu-ZnO-Pt@H-ZSM-5 catalyst remained securely anchored to the H-ZSM-5 membrane surface, showing minimal morphological changes [46].

Overall, ZMRs provide a promising strategy for enhancing the stability of catalytic systems for CO_2_ conversion. By combining catalytic transformation with selective molecular separation, these reactors can effectively regulate reaction environments, suppress catalyst deactivation pathways and improve long-term operational performance. Despite these advances, the long-term stability of membranes under high temperature and pressure must be carefully evaluated, particularly in the presence of steam and reactive intermediates. In addition, the reliable sealing and integration of membrane modules into reactors remain critical engineering challenges that influence overall reactor stability. Future research should focus on developing more robust membrane materials and optimizing reactor configurations for stable long-term operation.

#### Progress in process simulation of CO_2_ catalytic conversion

In addition to experimental studies, process modeling and simulation have played an important role in understanding and optimizing ZMR for CO_2_ conversion. Numerous studies have developed mathematical and computational models to evaluate the influence of membrane separation and reactor parameters on reaction performance and energy efficiency [47,123–125]. These modeling results can ultimately be used to design membrane reactors, interpret experimental data, guide the development of new experiments and identify key parameters for optimizing membrane reactor operation.

These modeling results can first directly demonstrate the advantages of membrane reactors over conventional FBRs in terms of CO_2_ conversion and product selectivity. For example, Barbieri *et al.* reported a simulation study on CO_2_ hydrogenation with simultaneous methanol/water removal in ZMR, where the results showed a higher methanol yield (13.7%) compared to FBR (5.8%) [126]. Larachi *et al.* developed a chemical reaction engineering model for a membrane reactor, which indicated that membrane reactors with *in situ* water removal are more efficient than FBR in DME synthesis [127]. Ateka *et al.* established a model to simulate the direct synthesis of DME in packed-bed membrane reactor (PBMR). The results demonstrated that the removal of water shifts the thermodynamic equilibrium, resulting in higher DME yield and CO/CO_2_ conversion compared to non-membrane systems. This model was further validated using laboratory-scale reactor experiments [128]. In addition, Avci *et al.* promoted process intensification by modeling a catalytic microreactor integrated with an SOD membrane, demonstrating its capability for efficient DME production [129]. Jung *et al.* also developed a mathematical model for a water/methanol-selective membrane reactor by incorporating reaction kinetics and gas permeation equations. The model was validated by comparison with experimental data from CO_2_ hydrogenation over a water-selective ZMR, confirming its reliability [62].

More recent simulation studies have systematically analyzed the influence of process and structural parameters, including membrane permeance, temperature, pressure, feed composition, sweep-gas flow condition, reactor geometry, etc. For example, Falco *et al.* evaluated membrane reactor performance using a one-dimensional (1D) non-isothermal model: to maximize DME yield, selectivity and CO*_x_* conversion, the temperature should be kept below 523 K to balance kinetics and thermodynamics, while high pressure enhances both reaction and permeation. A relatively low gas hourly space velocity (GHSV) is preferred, although excessively low flow rates may reduce productivity and require larger reactor volumes [130]. Samimi *et al.* proposed a spherical PBMR for methanol dehydration, which showed that this configuration could improve methanol conversion by ∼2.5% compared to conventional reactors [131]. Larachi *et al.* developed a 1D dynamic isothermal model for one-step DME synthesis, showing that water permeance above 10^−7^ mol m^−2^ s^−1^ pa^−1^ significantly enhances yield and selectivity, while higher CO_2_ feed fractions improve performance by suppressing the RWGS reaction [127].

Diban *et al.* identified an optimal membrane permeance range (0.5–1.2 × 10^−7^ mol m^−2^ s^−1^ pa^−1^) for simultaneously enhancing CO_2_ conversion and DME production, while noting that such ‘ideal’ membranes often suffer from limited hydrothermal stability. To compensate, they emphasized the importance of optimizing operating conditions and reactor configuration to minimize undesired mass transfer of reactants and intermediates [64]. Further analysis of sweep conditions showed that both sweep flow rate and recycle ratio play key roles, with CO_2_ conversion increasing significantly when the flow rate exceeds 0.18 mol CO h^−1^, as the efficient removal of water and methanol drives the reaction forward and maximizes DME yield [132].

Poto *et al.* employed a non-isothermal phenomenological 1D PBMR model to show that a maximum DME yield of 64% (via co-current sweep gas recycling) requires membranes with water permeance (4 × 10^−7^ mol m^−2^ s^−1^ pa^−1^) and selectivities of 50, 30 and 10 toward H_2_, CO_2_/CO and methanol, respectively [133]. In addition, membrane reactor performance also depends on the Damköhler number (Da), normalized gas permeance (θH_2_O or θMeOH) and pressure; increasing these parameters enhances CO_2_ conversion, and in methanol-selective systems also improves methanol selectivity [62]. Avci *et al.* developed a 2D isothermal model to analyze transport and reaction processes in membrane reactors, showing that higher syngas inlet velocity enhances steam removal and hydrogen transport, improving CO_2_ conversion and DME yield up to 15.8% and 17.4%, respectively [129].

In addition to evaluating reaction efficiency and the influence of key structural and operational parameters, recent process simulation studies have also focused on energy consumption analysis. For example, Hamedi *et al.* employed a pseudo-homogeneous model to simulate DME synthesis in a multitubular membrane reactor, demonstrating that membrane integration at 75 bar significantly reduces energy demand, lowering power, heating and cooling consumption by 1.5%, 44.5% and 69.4%, respectively, along with a 7.3% reduction in CO_2_ emissions [134]. Furthermore, Behloul *et al.* proposed optimizing reaction–separation matching using the Damköhler number to enhance synergy among reaction, heat transfer and membrane separation. By employing microchannel structures with high surface area-to-volume ratios, heat and mass transfer limitations are minimized, enabling near-isothermal operation, eliminating hot spots and reducing side reactions and energy losses [135].

Overall, existing process simulation studies demonstrate that factors such as membrane water selectivity and permeance, operating temperature and pressure, feed composition, sweep gas conditions and reactor configuration play critical roles in determining CO_2_ conversion, product selectivity and energy consumption in ZMRs. Therefore, systematic process modeling and parameter optimization are essential for guiding the design and industrial implementation of high-performance ZMRs. Nevertheless, compared with experimental studies, systematic process simulations for CO_2_ conversion in ZMRs remain relatively limited, particularly in terms of multiscale coupled modeling and full-process optimization, leaving substantial room for further development.

## FUTURE RESEARCH DIRECTIONS

As discussed earlier, the enhanced performance of ZMRs has been demonstrated in various chemical reactions involving CO_2_, such as CO_2_ hydrogenation to methanol and DME. Despite significant improvements in CO_2_ conversion and surpassing thermodynamic limits, these applications still require further investigation for several key reasons: (i) there are limitations in conversion efficiency and selectivity; and (ii) within the vast landscape of CO_2_ catalytic conversion, research has largely focused on methanol and DME production, while other areas, particularly the synthesis of DMC, remain relatively underexplored, highlighting the need for urgent further studies. Moreover, the design of high-performance membrane reactors relies on the coordinated advancement of catalysts, separation membranes and their coupled integration.

For catalysts, the in-depth development of catalysts is crucial for their rational use in ZMRs. Currently, Cu/ZnO/Al_2_O_3_ systems have been industrially applied in CO_2_ hydrogenation to methanol and represent one of the most mature catalytic systems. However, considering the vast landscape of CO_2_ catalytic conversion, there remains a strong need for the large-scale development of alternative catalysts with high availability and reproducibility. Moreover, ZMRs impose unique requirements that differ fundamentally from those in conventional catalytic systems. In ZMRs, the continuous removal of products such as H_2_O creates a dynamic and non-equilibrium reaction environment, which can significantly alter surface intermediates, reaction pathways and rate-determining steps. As a result, catalysts optimized in traditional FBRs may not exhibit optimal performance in membrane-coupled systems. Therefore, catalyst design for ZMRs should move toward environment-adaptive systems that maintain activity and stability under dynamically evolving conditions. A further challenge arises from the mismatch between catalyst and membrane operating conditions, where catalysts often require high temperatures for sufficient activity, while membranes demand conditions that ensure structural integrity and selective permeation. Therefore, the development of highly active low-temperature CO_2_ hydrogenation catalysts is particularly important to meet the requirements of ZMR systems. Recent reports of efficient catalysts operating at around 100°C have begun to address this gap [136]. These shifts from isolated catalyst optimization to system co-design represent a critical direction for advancing ZMR technology.

In ZMRs, membranes must simultaneously maintain high permeability, selectivity and structural integrity under high temperature and high pressure. In addition, achieving an optimal balance between hydrophilicity and framework stability remains a key challenge. Another important limitation relates to membrane microstructure and fabrication quality. Furthermore, scalability and reproducibility of membrane fabrication remain critical barriers to industrial deployment. The future research should focus on the rational design of membranes with optimized framework composition, controlled SARs and tailored extra-framework cations to regulate adsorption and transport behavior under reaction conditions. Meanwhile, advanced membrane architectures, including ultrathin, hierarchical and oriented zeolite membranes, are expected to significantly enhance permeation efficiency while maintaining selectivity. Moreover, new preparation strategies of zeolite membranes for ZMR applications should prioritize cost-effectiveness, environmental sustainability, universality, high repeatability and industrialization potential. A preparation strategy that meets the above requirements combined with microwave-assisted synthesis technology will hopefully lead to the scaling-up of ZMRs. Tubular or hollow fiber zeolite membrane should be actively developed for ZMR applications. Ultimately, progress in membrane materials must be integrated with reactor and process design considerations to ensure that the intrinsic advantages of zeolite membranes can be fully realized in practical ZMR systems for efficient CO_2_ conversion.

The core advantage of ZMRs lies in the dynamic coupling between catalytic reaction and selective separation. From a reaction engineering perspective, future efforts should focus on establishing effective communication between reaction kinetics and mass transfer processes. In an ideal ZMR, the rates of catalytic reaction and membrane permeation should be dynamically matched, such that the generation and removal of key species (e.g. H_2_O) occur in a coordinated manner [125]. However, in most existing systems, mismatches between reaction kinetics and membrane permeation lead to the accumulation of intermediates or by-products near catalytic sites, resulting in weakened equilibrium shifting effects and even catalyst deactivation. To overcome this limitation, future designs should emphasize spatial and kinetic coupling between catalysts and membranes. This includes minimizing diffusion distances through intimate catalyst-membrane contact, developing integrated architectures in which catalytic sites are directly anchored onto or embedded within the membrane structure. In addition, reactor optimization, such as tailoring flow configurations and pressure gradients, is essential to ensure uniform mass transfer and avoid concentration polarization. Importantly, the concept of rate matching (reaction vs permeation) should be introduced as a key design principle, enabling the system to operate under conditions where separation continuously reinforces reaction progress. Ultimately, achieving synergistic intensification in ZMRs requires a shift toward multiscale co-design, where catalyst properties, membrane transport characteristics and reactor configurations are simultaneously optimized to maximize coupling efficiency. This integrated approach provides a clear pathway toward the development of membrane reactors for efficient and stable CO_2_ conversion.

The practical deployment of ZMR for CO_2_ conversion is also constrained by challenges associated with process conditions, reactor design and scale-up. Process operation parameters play a decisive role in determining ZMR performance and stability [29]. For example, the trade-off between enhanced reaction kinetics and thermodynamic equilibrium at elevated temperatures and the potential formation of membrane defects must be carefully balanced. Similarly, elevated pressures enhance permeation driving force and reaction conversion but may exacerbate membrane defects and influence product selectivity. The interplay between feed composition, sweep gas flow rate and GHSV further complicates process optimization, as these parameters simultaneously affect reaction rates, permeation efficiency and catalyst stability. In addition, sweep gas and recycling operations are essential in CO_2_ hydrogenation processes, and the associated technical challenges must be systematically evaluated. Energy consumption related to heating, cooling and compression of sweep streams should also be carefully assessed to ensure process feasibility.

Expanded efforts in multiscale modeling and simulation, along with the integration of *in situ* characterization techniques, are needed to unravel the ‘black box’ of coupled catalytic and membrane processes. Such approaches will help guide the optimization of ZMR fabrication and improve long-term stability. From an engineering perspective, the design and configuration of membrane reactor modules must particularly address sealing issues, especially under harsh operating conditions (200–400°C, 20–40 bar and steam-rich environments), where reliable gas-tight sealing remains challenging. Furthermore, the scalable manufacturing of reactor assemblies continues to be a major bottleneck, requiring the development of automated, reproducible and cost-effective fabrication methods, as well as the use of sustainable and recyclable materials.

Looking forward, the advancement of ZMR technology will rely on the coordinated development of catalytic materials, membrane materials, reactor configurations and integrated process design, where sealing strategies, operating conditions and system-level energy management must be optimized in a synergistic manner. In particular, multiscale modeling and simulation are expected to play a crucial role in elucidating the complex coupling between reaction and separation processes, thereby guiding rational reactor design. In addition, the application of *in situ* and operation characterization techniques will enable a deeper understanding of the structural and functional evolution of ZMRs under realistic working conditions. Ultimately, bridging the gap between laboratory-scale demonstrations and industrial implementation requires not only advances in materials but also breakthroughs in module engineering, process optimization and system integration, paving the way for economically viable and stable CO_2_ conversion using ZMR technology.

## CONCLUSIONS

In summary, ZMRs represent a promising platform for overcoming thermodynamic limitations and enhancing the efficiency of CO_2_ catalytic conversion. While significant progress has been achieved, particularly in methanol and DME synthesis, key challenges remain in expanding reaction scope, improving selectivity and ensuring long-term stability. Future advancements should shift from isolated optimization of catalysts or membranes toward integrated system design, where catalytic activity, membrane transport properties and reactor configurations are synergistically matched. The development of environment-adaptive catalysts, high-performance and scalable zeolite membranes and dynamically coupled reaction–separation systems will be essential. In parallel, multiscale modeling, *in situ* characterization and engineering innovations in reactor design and sealing strategies must be strengthened to bridge the gap between laboratory research and industrial application. Overall, the rational co-design of materials and processes provides a clear pathway for realizing efficient, stable and economically viable CO_2_ conversion using ZMR technology.
